# Studies on spiro[4.5]decanone prolyl hydroxylase domain inhibitors†
†Electronic supplementary information (ESI) available. See DOI: 10.1039/c8md00548f


**DOI:** 10.1039/c8md00548f

**Published:** 2019-03-01

**Authors:** James P. Holt-Martyn, Anthony Tumber, Mohammed Z. Rahman, Kerstin Lippl, William Figg, Michael A. McDonough, Rasheduzzaman Chowdhury, Christopher J. Schofield

**Affiliations:** a Department of Chemistry , University of Oxford , Chemistry Research Laboratory , 12 Mansfield Road , Oxford , OX1 3TA , UK . Email: christopher.schofield@chem.ox.ac.uk

## Abstract

The 2-oxoglutarate (2OG) dependent hypoxia inducible factor (HIF) prolyl hydroxylases (PHDs) are targets for treatment of anaemia and other ischaemia related diseases. PHD inhibitors are in clinical trials; however, the number of reported templates for PHD inhibition is limited. We report structure–activity relationship and crystallographic studies on spiro[4.5]decanone containing PHD inhibitors. Together with other studies, our results reveal spiro[4.5]decanones as useful templates for generation of potent and selective 2OG oxygenase inhibitors.

## Abstract 2

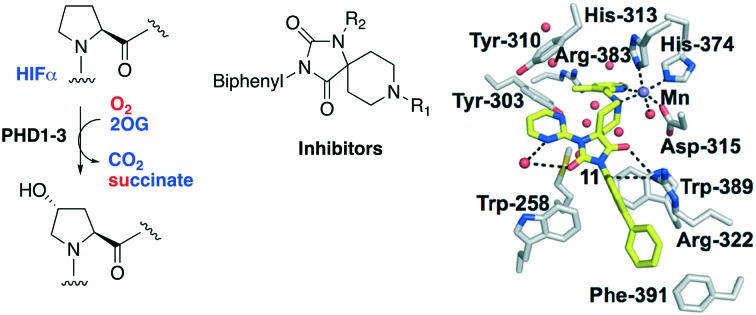
Structure–activity relationship and crystallographic studies on HIF prolyl hydroxylase inhibitors reveal spiro[4.5]decanones as useful templates for generation of potent and selective 2OG oxygenase inhibitors.

## 

The α,β-heterodimeric hypoxia-inducible transcription factors (HIFs) are of central physiological importance in the response to chronic limited oxygen availability in animals.^1^–^5^ HIF target genes include those encoding for multiple proteins of bio-medicinal importance, including erythropoietin (EPO), vascular endothelial growth factor (VEGF), and proteins involved in epigenetic regulation. Prolyl hydroxylation of HIF-α subunits signals for their degradation *via* the ubiquitin–proteasome system. Inhibition of the human HIF-α prolyl hydroxylases (PHD1-3) has the potential to mimic elements of the physiological hypoxic response. PHD inhibitors are in phase 3 clinical trials for the treatment of anaemia in chronic kidney disease, *via* upregulation of EPO (Fig. 1).^1^–^7^ All of the PHD inhibitors currently in the clinical trials bind to the active site Fe(ii) of the PHDs and compete with the 2-oxoglutarate (2OG) co-substrate.^7^,^8^ The extent to which the inhibitors compete with HIF-α, and maybe other PHD substrates varies.^7^,^9^,^10^ All of these PHD inhibitors have the potential to inhibit other human 2OG oxygenases, including other prolyl hydroxylases (*e.g.* procollagen and ribosomal prolyl hydroxylases).^7^ Many of the reported PHD inhibitors are structurally related to 2OG, and are relatively flat heteroaromatic compounds (Fig. 1).^9^–^11^ Thus, there is a desire to generate new types of PHD inhibitors, with improved potency and selectivity. In this regard, the PHD inhibitors reported by Vachal *et al.*, are of interest because of their chiral spirocyclic nature (Fig. 2, panel A).^12^,^13^

**Fig. 1:**
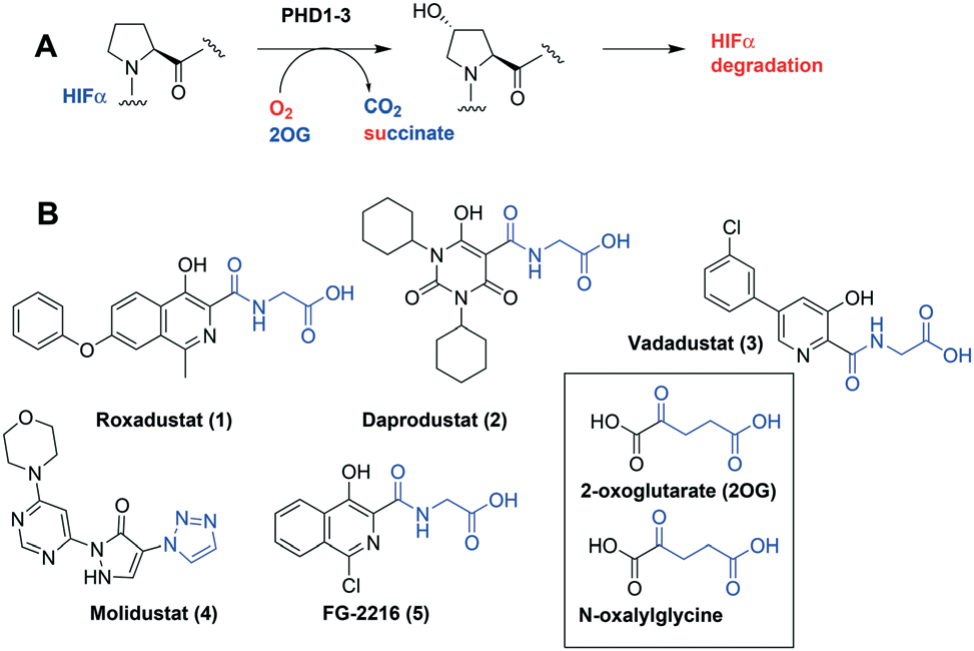
The HIF prolyl hydroxylases are therapeutic targets. A. Prolyl-4-hydroxylation of prolyl residue in hypoxia inducible factor α (HIF-α) subunits, signals for degradation *via* the ubiquitin proteasome system. 2OG, 2-oxoglutarate; PHD1-3, human prolyl hydroxylase enzymes 1–3; VHL-E3 ligase: the von Hippel Lindau protein (VHL) is the targeting component of a ubiquitin E3 ligase system. B. Examples of PHD inhibitors in clinical trials. Roxadustat (FG-4592, **1**), daprodustat (GSK1278863, **2**), vadadustat (**3**), and molidustat (BAY 85-3924, **4**).^6^,^7^ Structures of 2OG and *N*-oxalylglycine a broad-spectrum 2OG oxygenase inhibitor are shown.

**Fig. 2:**
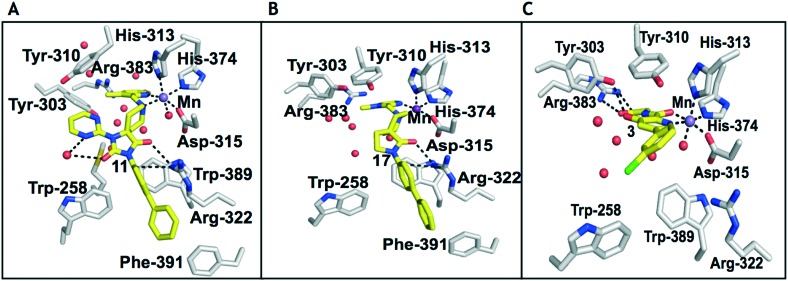
Comparison of views from crystal structures from PHD2_181–407_·Mn^II^ in complex with **11** (PDB ; 6QGV) (A), **17** (PDB ; 4JZR)^13^ (B) and **3** (PDB ; 5OX6) (C).^7^ Analysis of the binding modes of **11** & **17** reveal that they occupy the 2OG binding pocket and chelate the active site metal (Mn substituting for Fe) in a bidentate manner. Note the extent to which the PHD inhibitors project into the substrate binding pocket varies. In the case of the spiro[4.5]decanone inhibitors (A and B) the biphenyl ring group projects into an aromatic pocket formed by the side chains of Trp-258, Trp-389 and Phe-391, which are involved in substrate binding. The inhibitors also interact with the catalytically important residue Arg-322.^16^,^17^

The spirocyclic core of the spiro[4.5]decanone series has potential for expansion into both the substrate and 2OG binding pockets of the PHDs (Fig. 2), and is apparently amenable to development of PHD selective and, more generally, 2OG oxygenase inhibitors.^7^ Limited SAR has been reported for the spiro[4.5]decanones series by Vachal *et al.* and Deng *et al.*, and no details for the potential of the series for selective inhibition has been described.^12^,^13^ Here we report SAR and structural studies of spiro[4.5]decanone containing PHD inhibitors (**11–16**, **23–27**, **36–44**).

Prior analyses of truncated PHD2 in complex with a spiro[4.5]decanone inhibitor reveal a binding mode involving chelation of the active site metal (Mn(ii) was substituted for Fe(ii) to produce a complex stable for crystallization) *via* the tertiary amine of its piperidine ring and a pyridinyl nitrogen (Fig. 2).^13^ Chelation of the active site metal of 2OG oxygenases by a pyridine ring is well precedented, *e.g.* with pyridine carboxylate inhibitors.^14^ However, tertiary alkylamine chelation as observed for **17** is less well documented, a rare example being daminozide (HO_2_CCH_2_CH_2_CONHNMe_2_), which inhibits specific JmjC histone demethylases (KDMs) *via* chelation of its carbonyl oxygen and the tertiary amine group of its acyl hydrazide.^15^

There are three human HIF-α isoforms, with HIF-1α and HIF-2α having *N*- (NODD) and *C*-terminal (CODD) oxygen dependent degradation domains, each with a single prolyl hydroxylation site.^1^–^3^ Work with the HIF hydroxylases has revealed differences outside of the 2-OG CH_2_CH_2_CO_2_H binding pocket that can be exploited to develop potent inhibitors.^16^ We therefore began by investigating substitution of the piperidone ring of the spiro[4.5]decanone series.

1,3,8-Triazaspiro[4.5]decane-2,4-dione (**11**) was synthesised by the route of Vachal *et al.* (Fig. 3).^12^ Thus, piperidone (**6**) underwent Bucherer–Berg reaction to give **7**; consecutive Ullmann couplings gave **8** then **9**; formic acid mediated Boc deprotection gave **10**. Reductive amination gave the targeted compounds **11–16** (Fig. 3).

**Fig. 3:**
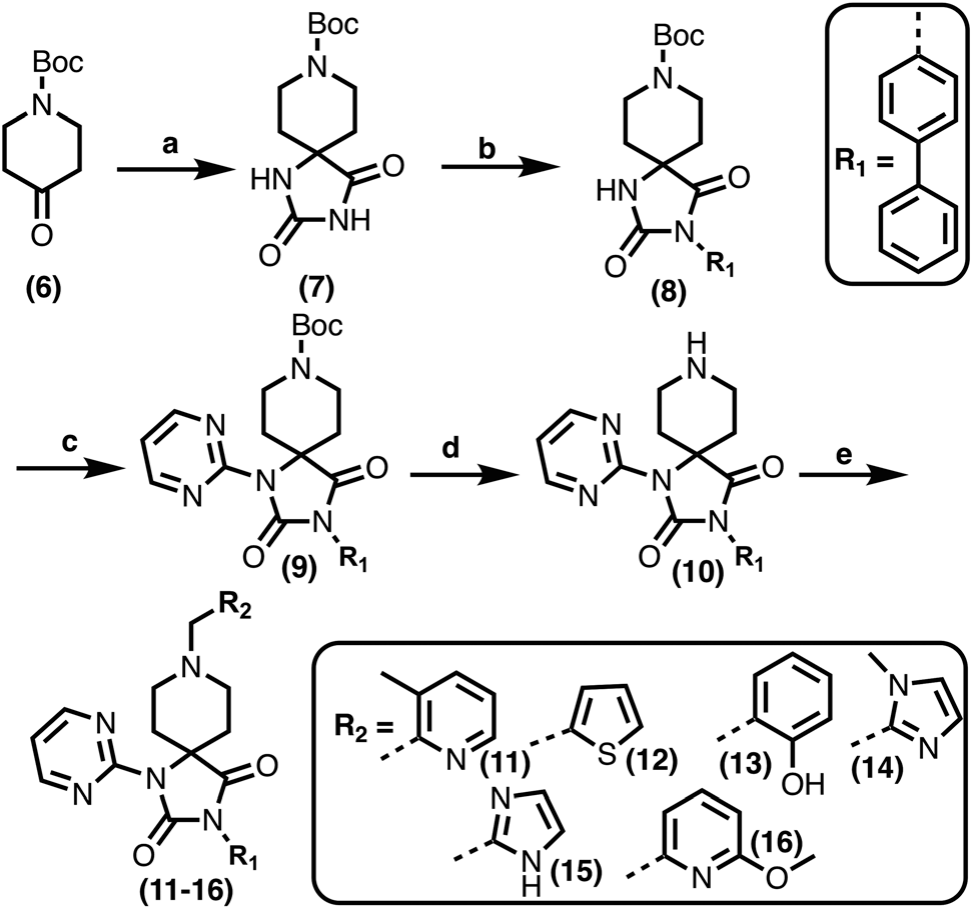
Synthesis of 3-([1,1′-biphenyl]-4-yl)-8-((aryl)-1-(pyrimidin-2-yl)-1,3,8-triazaspiro-[4.5]-decane-2,4-dione compounds (**11–16**) to investigate the role of the pyrimidine group in tPHD2 inhibition. Conditions: a) KCN, NH_4_CO_3_, EtOH : H_2_O (1 : 1), 60 °C; b) CuI, K_2_CO_3_, 4-iodobiphenyl,*N*,*N*-dimethyl-ethylenediamine, ascorbate, DMF : CH_3_CN (1 : 1), reflux; c) 2-iodopyrimidine, CuI, TMHD, Cs_2_CO_3_, DMF : CH_3_CN (1 : 1), reflux; d) HCOOH, rt, 3 h; e) RCHO, NaBH(OAc)_3_, HCOOH. R_1_ = biphenyl.

**11–16** were then assayed using mass spectrometry (MS) for inhibition of the catalytic domain of truncated PHD2 (tPHD2, residues 181–426) and (in selected cases) PHD3, employing either human HIF-1α CODD or NODD peptide sequences (Table 1). Details of the cellular activities of **11–16** and related compounds will be published elsewhere.

**Table 1:** SAR of spiro[4.5]decanone containing inhibitors. Inhibitors were screened against tPHD2 with HIF-1α CODD and NODD substrates and PHD3 HIF1-α CODD substrate using a RapidFire mass spectrometer. Standard error of the mean (*n* = 3)

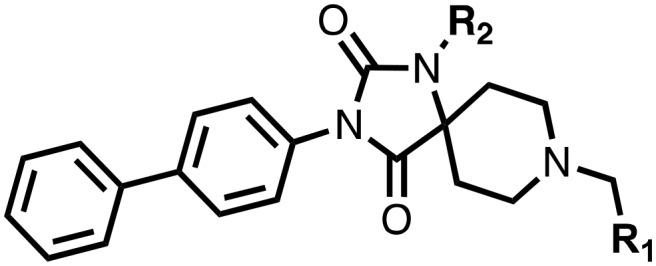				
Compound	R_1_	R_2_	PHD2 with HIF1-α CODD IC_50_ μM	PHD2 with HIF1-α NODD IC_50_ μM
**10**	H--	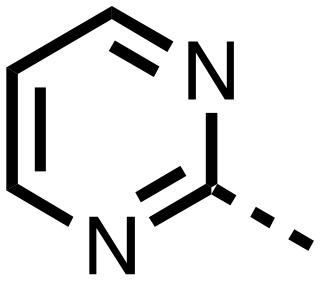	>25	>25
**11**	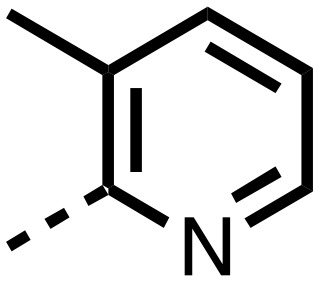	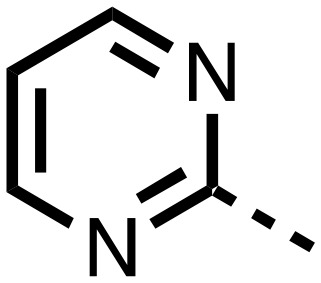	0.253 ± 0.047	0.127 ± 0.056
**12**	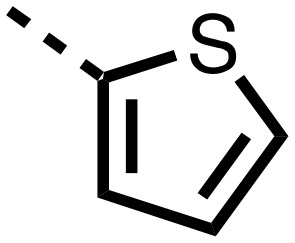	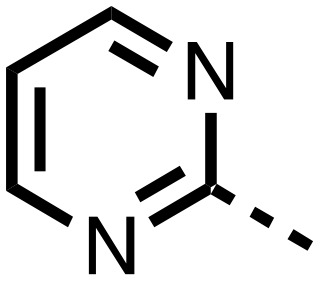	>25	>25
**13**	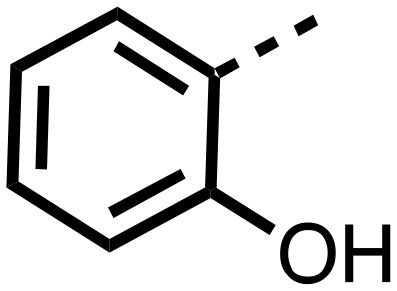	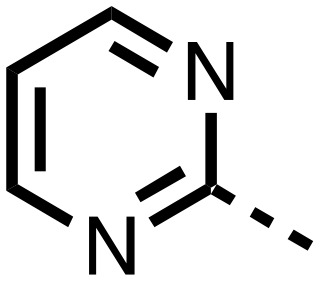	>25	>25
**14**	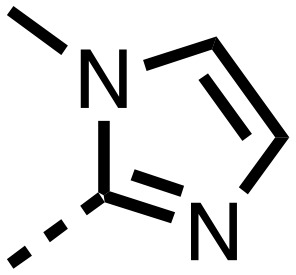	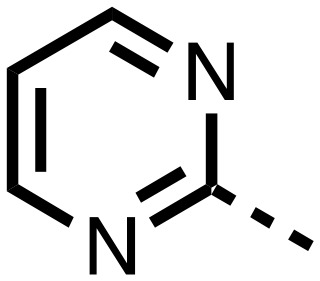	0.143 ± 0.097	0.049 ± 0.005
**15**	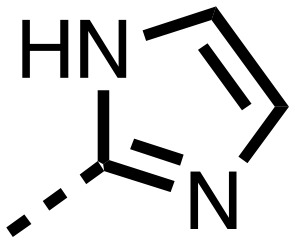	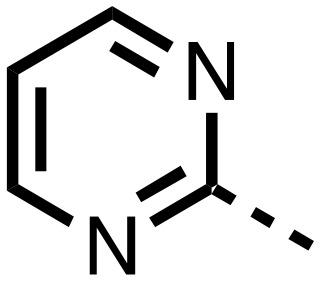	0.215 ± 0.019	0.070 ± 0.021
**16**	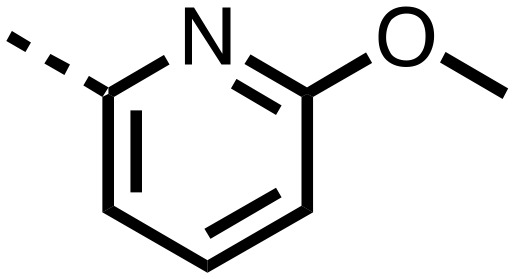	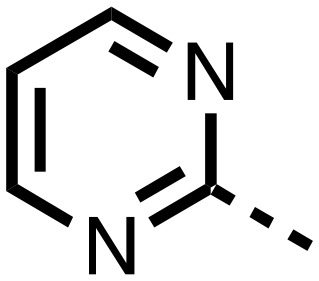	1.21 ± 0.226	0.905 ± 0.417
**23**	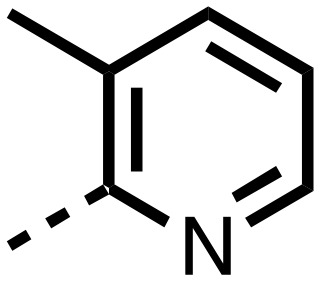	H--	0.741 ± 0.340	0.427 ± 0.122
**24**	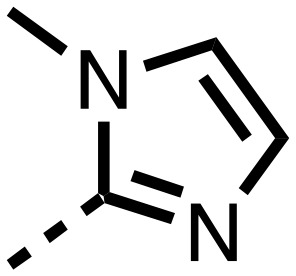	H--	0.151 ± 0.035	0.063 ± 0.016
**26**	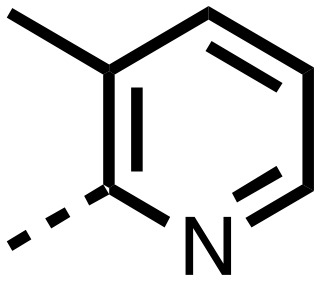	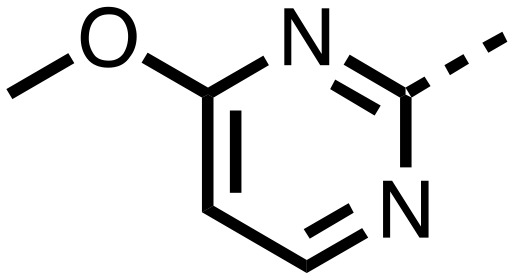	0.289 ± 0.011	0.055 ± 0.015
**27**	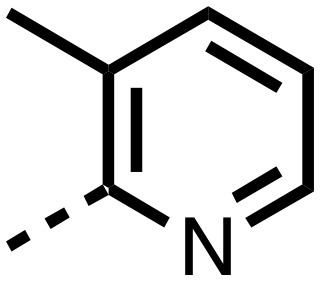	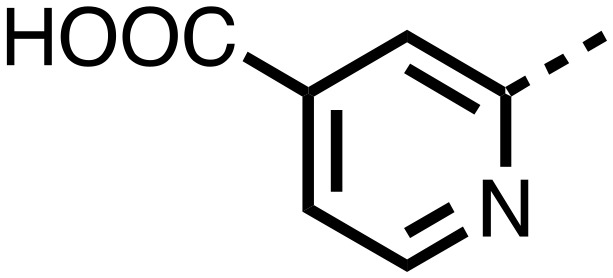	0.380 ± 0.064	0.094 ± 0.032

The inhibition results with **11–16** support the potential of the series for potent PHD inhibition and reveal potential for optimization. They reveal the importance of a chelating group at the 2-position of the pyridine ring or equivalent (the thiophene (**12**) and phenol (**13**) derivatives were inactive), and potential for variation in the metal chelating rings. Thus, the *N*-methyl imidazole (**14**) and imidazole (**15**) derivatives maintained potent inhibition comparable to (or better for the *N*-methyl imidazole (**14**)) the parent 3-methyl pyridine (**11**).^12^

To inform the SAR work, crystal structures of tPHD2 in complex with our 1,3,8-triazaspiro[4.5]decane-2,4-dione compound series were pursued. We obtained a structure of tPHD2 in complex with **11** and Mn^II^ (substituting for Fe^II^) (Fig. 2, S1, S2 and S4 and Table S3†). The new structure reveals that **11** makes similar interactions with PHD2 to those observed for **17**.^13^ The 3-methyl pyridine ring of **11** penetrates into the 2OG binding pocket.^7^,^8^ The active site metal is chelated by the tertiary amine of the piperidine ring and 3-methyl pyridinyl nitrogen of **11**. The biphenyl substituent of **11** is positioned in an aromatic pocket formed by the Trp-258, Trp-389 and Phe-391 side-chains, with which it makes hydrophobic/π-stacking interactions. The terminal phenyl ring of the biphenyl substituent of **11** is positioned to form a cation π-interaction with the catalytically important residue Arg-322.^17^,^18^ Arg-322 is also positioned to hydrogen bond to the imidazolidine-2,4-dione ring of **11** (Fig. 2, panel A). The pyrimidine ring of **11**, which is not present in **17**, is positioned to form a π-stacking interaction with the phenol ring of Tyr-310. The presence of the pyrimidine ring of **11** also apparently changes the conformations of catalytically important residues, including Tyr-303 and Tyr-310, compared to their conformations as observed in the structures of tPHD2 in complex with the close 2-OG analogue *N*-oxalylglycine (NOG) (Fig. S1, panel F†) or 2,8-diazaspiro[4.5]decan-1-one **17** (Fig. 2, panel B).^13^,^17^,^18^

From the initial work on the spiro[4.5]decanone series, it was unclear whether the pyrimidine ring of **11** is required for PHD2 inhibition. A series of analogues of **11** (Fig. S3, panel A†), wherein the pyrimidine ring was removed or modified, based on interactions observed in the PHD2:**11** structure, (Fig. S3, panel A†), were therefore prepared.

**23–26**, were synthesised using the same route as before (Scheme S1†). The inhibition results with these derivatives reveal the pyrimidine ring has a minor role in enabling potent tPHD2 inhibition, *i.e.* the non-pyrimidine containing analogues **23** are only somewhat weaker compared to **11** (IC_50_**11**, 0.253 μM; **23**, 0.741 μM). The substituted pyridyl analogues, such as **27**, were as active as **11** (IC_50_**11**, 0.253 μM; **26**, 0.289 μM; **27**, 0.333 μM). Previous work has shown that pyridine carboxylates can bind in the 2-OG pocket, but this seems unlikely to be the case for the pyridine carboxylate ring of **27**, as shown by our crystallographic analysis of **11** (Fig. 2 panel A, S1 and S2†).^14^

We then investigated the importance of the imidazol-idine-2,4-dione ring of **11** using compounds **36–44** (Fig. S3, panel B†). It was envisaged that replacement of the imidazolidine-2,4-dione ring with an amide group might fulfill the requirement for a linker of appropriate length between the elements binding in the 2-OG binding and aromatic ring binding pockets (Fig. 2, panel A). Substituents interacting with both the 2-OG binding and ‘aromatic’ pockets were varied in **36–44**. For the aromatic pocket binding groups, either 4-biphenyl or the 4-phenylbenzyl groups were used. For the heteroaromatic metal binding substituent, 3-methyl pyridine and phenol substituents were chosen as they had previously manifested different levels of inhibition (Table 1).

The 3-hydroxypyridine derivatives **38**, **41** and **44** were produced because analysis of the PHD2 structure in complex with **11** (Fig. 2, panel A) suggested that replacing the methyl group of **11** with a hydroxyl group may form additional interactions with PHD2, *via* hydrogen bonding to either Tyr-310 or Tyr-303 (Fig. 2, panel A). To prepare **36–44**, the requisite carboxylic acids were coupled with the requisite amines to give **29**, **30**, **32**, which underwent Pd catalysed deprotection to provide the amines **33–35**. Application of the reductive amination conditions developed for the 1,3,8-triazaspiro[4.5]-decane-2,4-dione series, gave the targeted compounds **36–44** (Scheme S2†). The imidazole2,4-dione analogues **36–44** were inactive, implying an important role for the imidazolidine-2,4-dione core and a relatively rigid linker between elements binding in the aromatic and 2OG binding pockets (Table S1†).

We carried out initial studies on the selectivity of the spiro[4.5]decanone series. The assay results with tPHD2 and either HIF-1α NODD or CODD as substrates showed similar results in terms of rank order (Table 1). However, the IC_50_ values for NODD were in general lower than for CODD, perhaps reflecting its lower efficiency as a tPHD2 substrate.^2^–^4^ Several of the compounds were tested as PHD3 inhibitors and were observed to inhibit PHD3 catalysed either HIF-1α CODD hydroxylation at similar levels observed for PHD2 inhibition (PHD3 IC_50_ values: **11**, 3.95 ± 0.75 μM; **23**, 1.05 ± 0.04 μM; **24**, 0.219 ± 0.025 μM). This observation suggests that the spiro[4,5]decanone core is not, at least intrinsically, PHD2 isoform selective. However, the differences in IC_50_ values for tPHD2 and PHD3 also suggest that modification of the spiro[4,5]decanone core might be exploited for development of PHD isoform selective inhibitors. However, care should also be taken when assessing the results with isolated HIF-α fragments and initial kinetic assays as employed here, which may not translate to a cellular context. Further evidence that spiro[4.5]decanones have potential as useful 2OG oxygenase inhibitors comes from selectivity studies on compounds **11–16**, **23–27** with isolated forms of two other human oxygenases, *i.e.* the HIF-α asparaginyl hydroxylase factor inhibiting HIF (FIH) and a JmjC *N*^ε^-methyl lysine histone demethylase (KDM4).^7^ For all the tested compounds either no (*i.e.* IC_50_ >25 mM) or weak (**26** with FIH; **11**, **14** with KDM4A) inhibition was observed (Table S2†). The lack of activity *versus* FIH is important because this enzyme has non HIF-α substrates.^19^ Thus, spiro[4.5]decanone derivatives have potential use in dissecting the roles of individual HIF-α hydroxylases.

Together with previous work, our overall results reveal spiro[4.5]decanones as having considerable potential for inhibition of the PHDs.^12^ Initial selectivity studies have demonstrated the potential for selective 2-OG oxygenase inhibition by spiro[4.5]decanones series (Table S2†). More generally, the results reveal the potential for combining active site metal chelation with 3-dimensional elements to enable selectivity in the inhibition of 2-OG oxygenases.

## Conflicts of interest

C. J. S. is a co-founder of a company, ReOX, which aims to exploit basic science discoveries about the hypoxic response for therapeutic benefit.

## Supplementary Material

Supplementary information
