# Supplementary material for: Studies on spiro[4.5]decanone prolyl hydroxylase domain inhibitors
Source: Medchemcomm. 2019 Mar 1;10(4):500–4. doi: 10.1039/c8md00548f (PMC6482412; doi:10.1039/c8md00548f)
Supplement: Supplementary file 1 [file MD-010-C8MD00548F-s001.pdf]

Electronic Supplementary Material (ESI) for MedChemComm.
This journal is © The Royal Society of Chemistry 2019

Supporting Information:

Studies on Spiro[4.5]decanone Prolyl Hydroxylase Domain Inhibitors

James P. Holt-Martyn,[a] Anthony Tumber,[a] Mohammed Z Rahman,[a] Kerstin Lippl,[a] William Figg Jr,[a] Michael A. McDonough,[a] Rasheduzzaman Chowdhury [a] and Christopher J Schofield*[a]

Department of Chemistry, University of Oxford, Chemistry Research Laboratory, 12 Mansfield Road, Oxford, OX1 3TA, United Kingdom.

*To whom correspondence should be addressed. Email: christopher.schofield@chem.ox.ac.uk. Telephone: +44 (0)1865 285 000. Fax: +44 (0)1865 285 002. Address: Chemistry Research Laboratory, 12 Mansfield Road, Oxford, OX1 3TA, United Kingdom.

Contents

Figures.. 1

Figure S1: Comparison of views from crystal structures of PHD2 in complex with **11** (PDB ID 6QGV), **17** (PDB ID 4JZR), **3** (PDB ID 5OX6), **barbiturate** (PDB ID 5OX5), **IOX4** (PDB ID 5A3U), and NOG and the HIF-1α CODD substrate.. 1

Figure S2: Surface representation from a crystal structure of PHD in complex with **11**.................. 2

Figure S3: Graphical representation of the SAR performed on spiro[4.5]decanone containing PHD inhibitors.. 3

Figure S4: Stereo-view representation of the PHD2.**11** complex active site showing OMIT Fo-Fc map (contoured to 3.0 σ) around the ligand.. 3

Schemes.. 4

Scheme S1: Route for the synthesis of 3-([1,1'-biphenyl]-4-yl)-8-((pyridinyl)methyl)-1-(aryl)-1,3,8-triazaspiro[4.5]decane-2,4-dione series (**23**-**27**).. 4

Scheme S2: Route for the synthesis of analogues (**36**-**44**).. 4

Inhibition Tables.. 5

Table S1: SAR of analogues (**36**-**44**) investigating the role of the imidazolidine-2,4-dione ring screened against $tPHD2_{181\text{-}426}$ with HIF-1α CODD substrate.. 5

Table S2: Studies on the selectivity of spiro[4.5]decanone containing inhibitors. Screened against FIH with HIF-1α CAD peptide (D788 - L822) and KDM4A with H3(1 - 15) K9Me3.................... 6

General Experimental Methods.. 7

Preparation of tPHD2 (181 - 426 residues).. 7

X-ray Crystallography.. 8

Table S3: Crystallographic data processing and refinement statistics.. 9

RapidFire-MS PHD2 hydroxylation assay.. 10

Synthesis.. 12

General Considerations For Synthesis.. 13

General Procedures…… 12

Experimental Methods for compounds **7 - 44**…… 14

Representative NMR spectra…… 45

References…… 57

Figures

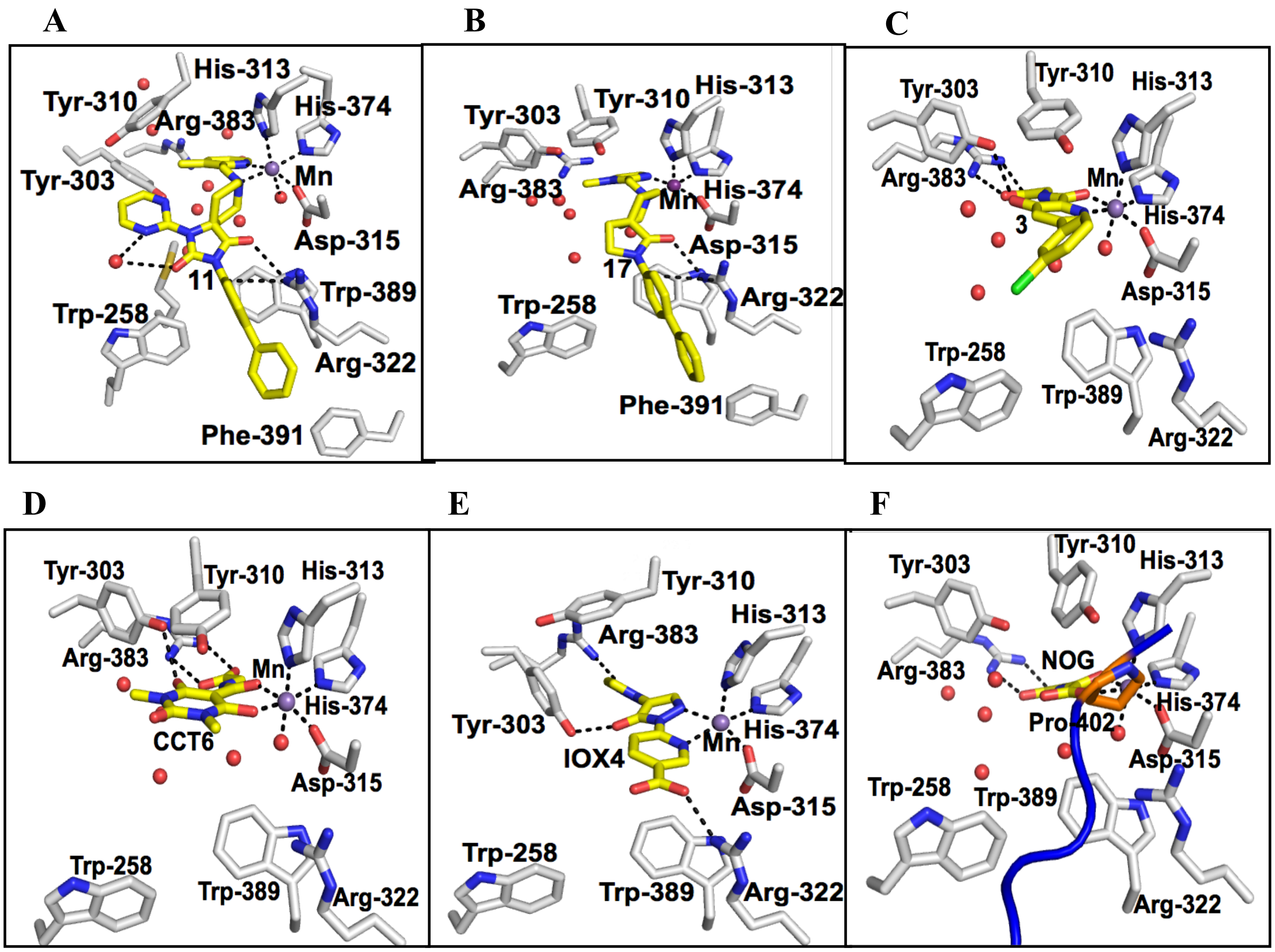

Figure S1: Comparison of views from crystal structures of PHD2.Mn in complex with (A) **11**, (B) **17** (PDB 4JZR),[1] (C) **3** (PDB 5OX6),[2] (D) **CCT6** (PDB 5OX5),[2] (E) **IOX4** (PDB 5A3U),[3] and (F) NOG and the HIF-1α NODD substrate (PDB 5L9V).[4]

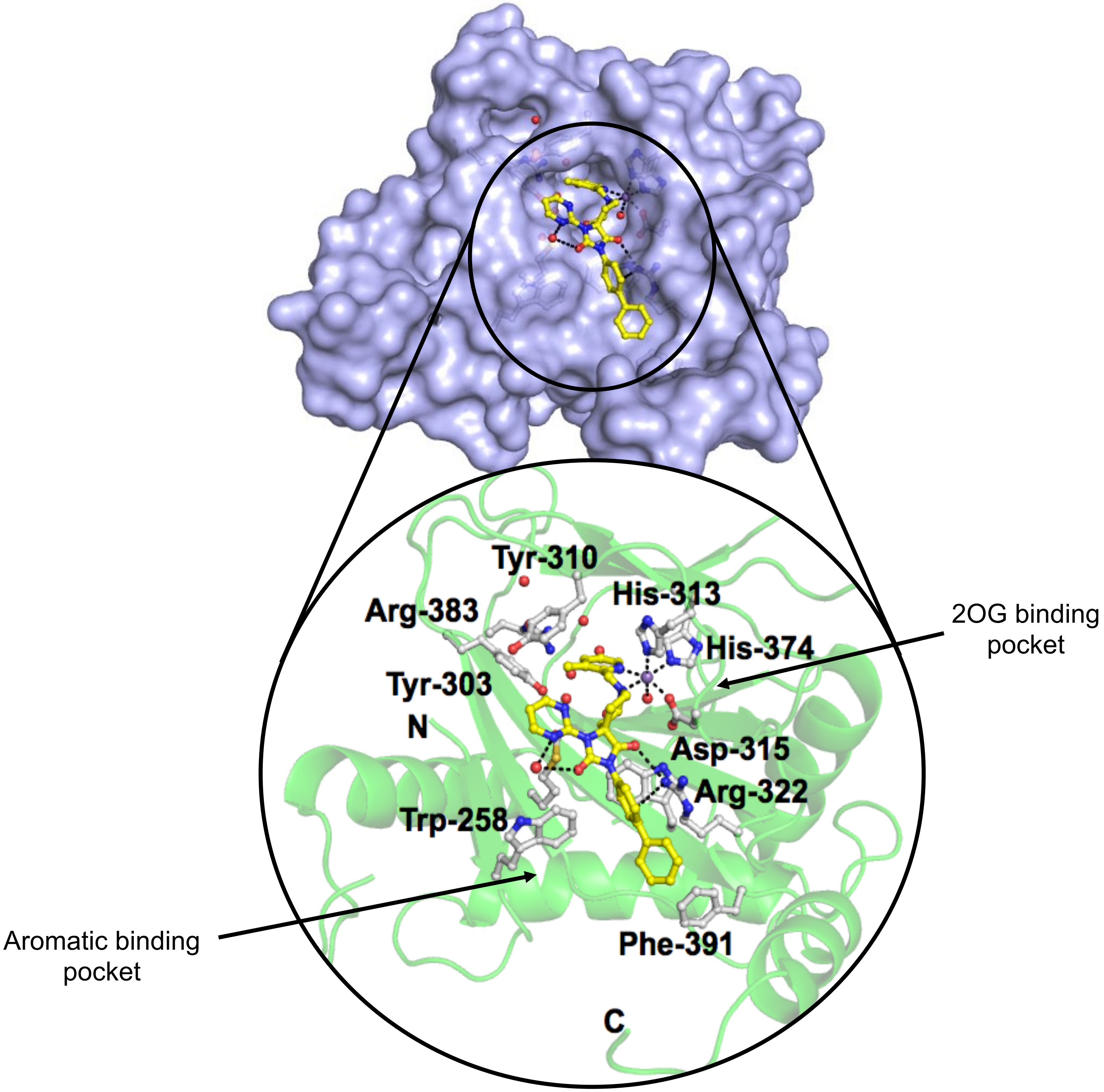

Figure S2: Surface representation from a crystal structure of $PHD2_{181\text{-}407}$.Mn in complex with **11**. Note that **11** occupies both the 2-OG binding pocket and an aromatic pocket formed by the side chains of Trp-258, Trp-389 and Phe-391.

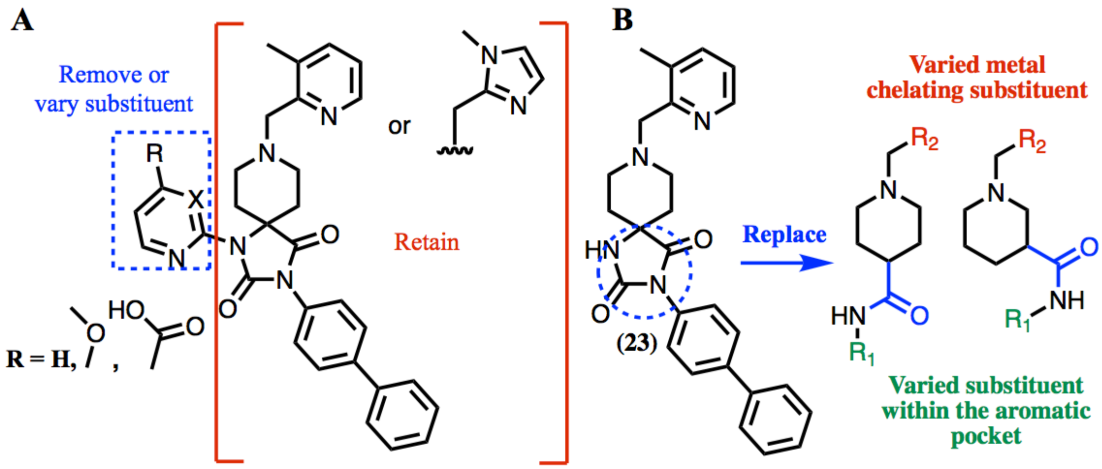

Figure S3: Graphical representation of the SAR study performed on spiro[4.5]decanone containing PHD inhibitors. (**A**) SAR investigations on the role of the pyrimidine ring; (**B**) SAR investigations on the role of the imidazolidine-2,4-dione ring.

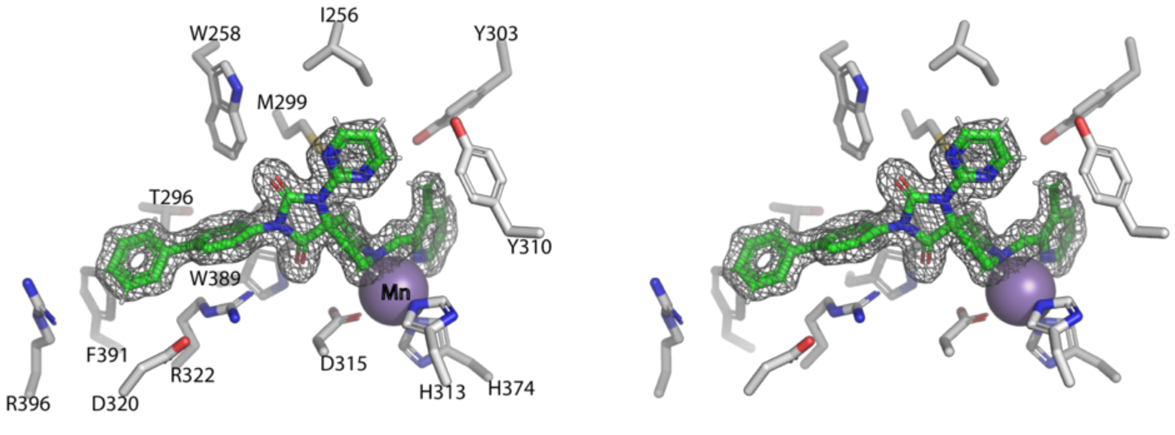

Figure S4: Stereo-view representation of the $PHD2_{181\text{-}407}$.Mn.**11** complex active site showing the OMIT F_o-F_c map (contoured to 3.0 σ) around the ligand.

Synthetic Schemes

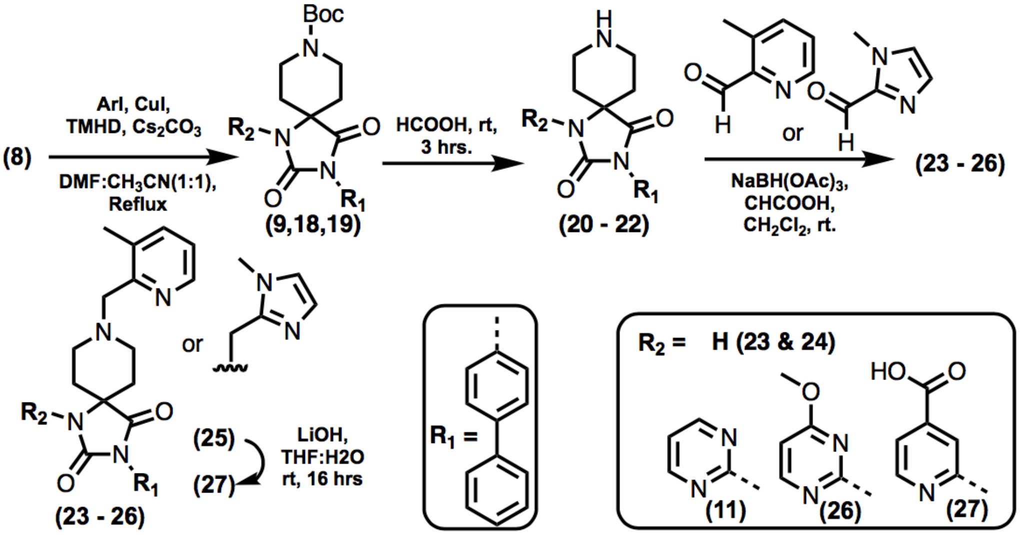

Scheme S1: Route for the synthesis of the 3-([1,1'-biphenyl]-4-yl)-8-((pyridinyl)methyl)-1-(aryl)-1,3,8-triazaspiro-[4.5]decane-2,4-dione series (**23 - 27**) which were prepared in order to investigate the role of the pyrimidine ring in PHD2 inhibition.

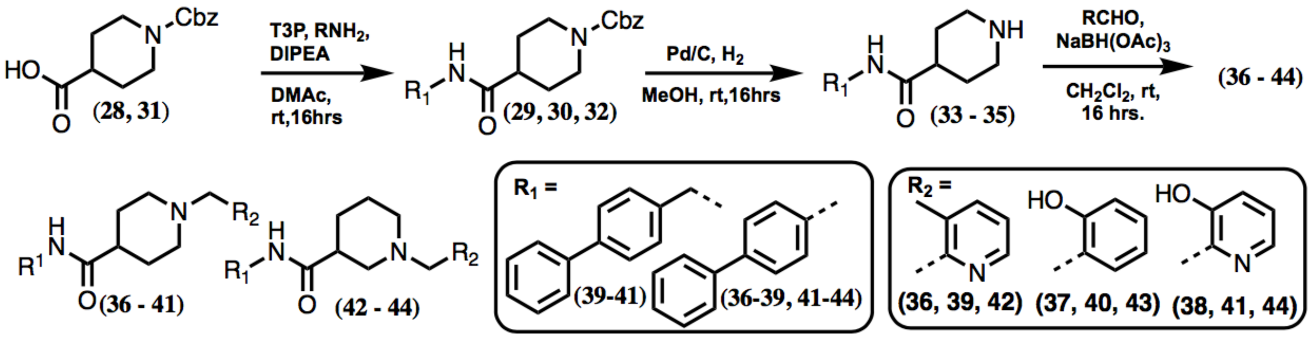

Scheme S2: Route for the synthesis of analogues (**36-44**) which were prepared in order to investigate the role of the imidazolidine-2,4-dione core of **23**.

Inhibition Tables

Table S1: SAR of analogues (**36-44**) which were prepared in order to investigate the role of the imidazolidine-2,4-dione ring in PHD inhibition. Compounds were screened against $PHD2_{181-426}$ (tPHD2) with a HIF-1α CODD substrate; the assay employed a RapidFire mass spectrometer. Standard error of the mean (n=3). See Experimental Methods for details.

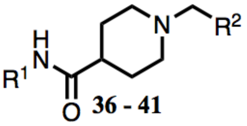

36 - 41

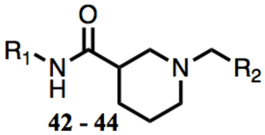

42 - 44

Cpd	R^1	R^2	IC_{50} µM
**36**	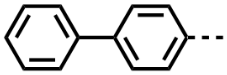	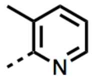	**>25**
**37**	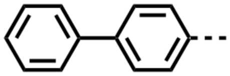	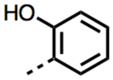	**>25**
**38**	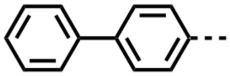	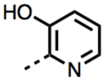	**>25**
**39**	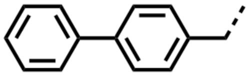	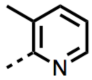	**>25**
**40**	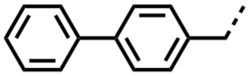	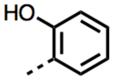	**>25**
**41**	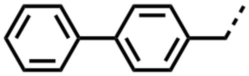	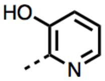	**>25**
**42**	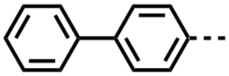	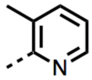	**>25**
**43**	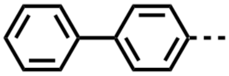	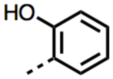	**>25**
**44**	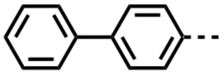	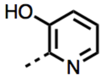	**>25**

Table S2: Studies on the selectivity of spiro[4.5]decanone containing inhibitors. Compounds were screened against FIH with the HIF-1α CAD peptide D788 - L822 and KDM4A with H3(1 - 15) K9Me3. Both assays employed the RapidFire-MS sampling machine. See Experimental Methods for details. [2]

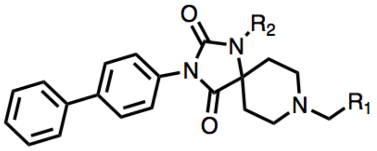

Cpd	R^1	R^2	FIH with HIF-1 CAD peptide D788 - L822 IC_{50} µM	KDM4A with H3(1 - 15) K9Me3 IC_{50} µM
11	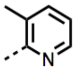	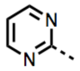	>25	**4.69**
12	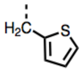	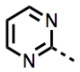	>25	>25
10	**H**	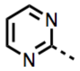	>25	>25
13	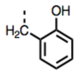	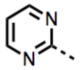	>25	>25
14	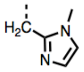	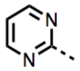	>25	**12.35**
15	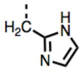	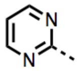	>25	>25
16	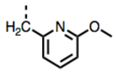	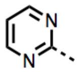	>25	>25
23	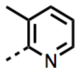	**H**	>25	>25
24	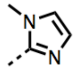	**H**	>25	>25
26	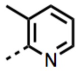	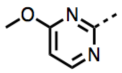	**21.60**	>25
27	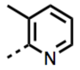	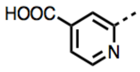	>25	>25

General Experimental Methods

Preparation of tPHD2$_{181-426}$

In brief, cDNA encoding for the catalytic domain of tPHD2 (tPHD2$_{181-426}$) was cloned into the pET28a(+) or pET24a(+) vectors (Novagen), as reported,[5] to enable production of tPHD2$_{181-426}$ protein with/without an N-terminal His$_{6\text{-tag}}$. The tPHD2$_{181-426}$ encoding construct was transformed into the *E. coli* BL21 DE3 cell line and protein production was induced with 0.5 mM isopropyl-b-D-thiogalactosidase (3–5 hr at 28℃). Cells were harvested and lysed by sonication in 20 mM Tris-HCl (pH 7.0) and 0.3 M NaCl; soluble protein (about 5% total soluble extract) was purified by immobilized Ni^{2+} affinity chromatography using pentadentate Tris-carboxymethyl ethylene diamine resin followed by cleavage of the His6-tag by thrombin (or alternately by cation exchange chromatography) with a final purification by gel filtration chromatography. The protein was exchanged into 50 mM Tris-HCl buffer (pH 7.5) and concentrated to % 40 mg/ml. The protein was of > 95% purity, as determined by SDS-PAGE analysis and mass spectrometry analysis.

X-ray Crystallography

Crystals of tPHD2$_{181\text{-}407}$ in complex with **11** were grown in 2.0 M ammonium sulfate, 5% ($^{v}/_{v}$) iso-propanol and were cryoprotected by transferring to a solution of mother liquor supplemented with 25% ($^{v}/_{v}$) glycerol. Data were collected at 100K using synchrotron radiation at the Diamond Light Source (DLS) beamline I03 and were autoprocessed using DIALS and SCALA.[6,7] The structure was solved by molecular replacement using PHASER (search model PDB ID 4BQX) and refined using PHENIX-refine.[8,9] Iterative cycles of model building in COOT and refinement proceeded until the R_{cryst}/R_{free} values no longer reduced or converged.[10]

Table S3: Crystallographic data processing and refinement statistics for the structure of PHD2$_{181\text{-}407}$.Mn in complex with **11**.

**PDB ID**	6QGV
**Data Collection**	
Beamline (Wavelength, Å)	0.97625
Detector	Dectris Pilatus 6M-F
Data processing software	DIALS, SCALA
Space Group	*H*32
Cell dimensions a, b, c (Å)	120.06, 120.06, 86.56
α, β, γ (°)	90, 90, 120
No. of molecules/ ASU	1
Resolution (Å)	60.03-1.40 (1.48-1.40)*
No. of unique reflections	47060 (6828)*
Completeness (%)	100 (100)*
Redundancy	19.2 (18.7)*
R_{sym}**	0.060 (1.646)*
Mean I/(I)	19.0 (2.1)*
Wilson *B* value ($Å^2$)	20.7
**Refinement**	
R_{factor}	0.157
R_{free}	0.172
R.m.s. deviation	
Bond length, (Å)	0.014
Bond angle, (°)	1.397
Ramachandran plot	
Most favoured regions	98.45
Additionally allowed regions	1.55
Disallowed regions	0.00

*Highest resolution shell shown in parenthesis.
**$R_{sym} = \sum|I-\langle I\rangle|/\sum I$, where I is the intensity of an individual measurement and $\langle I\rangle$ is the average intensity from multiple observations.
‡$R_{factor} = \sum_{hkl}||F_{obs}(hkl)| - k\,|F_{calc}(hkl)||/ \sum_{hkl}|F_{obs}(hkl)|$ for the working set of reflections; R_{free} is the R_{factor} for ~5% of the reflections excluded from refinement.
Polypeptide chain in parenthesis.
Note: The high redundancy data for the structure contributes to an exaggerated R_{sym} in the highest resolution bin – the I/σI value in this case is a more reasonable measure of the data quality.

RapidFire-MS PHD2 hydroxylation assays

Inhibition of PHD2 enzyme activity was assessed by mass spectrometry. The enzyme assay monitors $tPHD2_{181\text{-}426}$ catalysed turnover of a *C*-terminal oxygenase dependent domain peptide substrate (CODD) DLDLEMLAPYIPMDDDFQL (with a *C*-terminal amide) and appearance of the hydroxylated peptide product (hydroxylation of proline 564) with a typical incubation time of 15 minutes.[2] Assays were performed in 50 mM Tris.Cl pH 7.8, 50 mM NaCl, titrations of compounds for IC_{50} determinations (3-fold and 11-point IC_{50} curves) were performed using an ECHO 550 acoustic dispenser (Labcyte) and dry dispensed into 384-well polypropylene assay plates. The final assay concentration of DMSO was kept constant at 0.5% (v/v). $tPHD2_{181\text{-}426}$ was at a concentration of 300 nM in the assay buffer (20 μM ferrous iron sulfate, 200 μM L-ascorbic acid, 10 μM CODD or NODD peptide and 20 μM 2-oxoglutarate) and 25 μl was dispensed across each 384-well assay plate. $tPHD2_{181\text{-}426}$ was allowed to equilibrate with compounds for 15 minutes; the reaction was then initiated by addition of 25 μl of substrate.[2] Enzyme reactions were allowed to proceed for 15 minutes and the reaction terminated by addition of 10% ($^{v}/_{v}$) formic acid (5 μl). Assay plates were then transferred to a RapidFire RF360 sampling robot (Agilent) connected to an Agilent 6530 quadrupole-time-of-flight (Q-TOF) mass spectrometer. Assay samples were aspirated under vacuum and loaded onto a *C*4 solid phase extraction (SPE) cartridge. After loading, the C4 SPE cartridge was washed with 0.1 % ($^{v}/_{v}$) formic acid in water to remove non-volatile buffer salts. The peptide was then eluted from the SPE with 85% acetonitrile, 15% water containing 0.1% ($^{v}/_{v}$) formic acid into the mass spectrometer. Peptide charge states were monitored in the positive ion mode. Ion chromatogram data were extracted for

the +2 charge state and peak area data integrated using RapidFire Integrator software (Agilent). The % conversion of the CODD peptide substrate to the +16 hydroxylated peptide was calculated using the equation:

% conversion = 100 x hydroxylated / (hydroxylated + non-hydroxylated peptide): IC_{50} data were determined from non-linear regression plots using GraphPad prism 6.0. The level of +16 (methionine residue oxidation) as observed in the no enzyme control was around 4 - 5%. All data were normalized to a no enzyme control.[2]

General Considerations for Synthesis

All reactions involving moisture-sensitive reagents were carried out under a nitrogen atmosphere using standard vacuum line techniques. Glassware was oven dried and cooled under nitrogen before use. Commercial anhydrous solvents used in reactions and HPLC grade solvents were employed for work-up and chromatography. Water was purified using an Elix UV-10 system. Aqueous solutions were made using de-ionized water. Thin layer chromatography (TLC) was carried out using Merck (Darmstadt, Germany) silica gel 60 F254 TLC plates. TLC visualisation was carried out under UV light and stained with one of three stains; ninhydrin, potassium permanganate, or anisaldehyde. Chromatographic purifications were carried out using a Biotage® (Uppsala, Sweden) Isolera One or Biotage® SP4 flash purification system, using Biotage® pre-packed SNAP columns. Reactions were monitored using an Agilent (Cheshire, UK) 1200 series, 6120 quadrupole LC-MS system using a Merck Chromolith® Performance RP-18 HPLC column. Deuterated solvents were obtained from Sigma-Aldrich, and ^{1}H NMR spectra were obtained using Bruker AVANCE AVIII HD 400 nanobay (400 MHz) machine or a machine Bruker AV500 (500M Hz) with a ^{13}C cryoprobe. All signals are described in δ ppm with multiplets being denoted as singlet, doublet, triplet, quartet, and multiplet using the abbreviations s, d, t, q, and m, respectively. Chemical shifts in presented NMR spectra were referenced using residual solvent peaks with coupling constants, *J*, reported in hertz (Hz) to an accuracy of 0.5 Hz. For high-resolution mass spectrometry (HR-MS), a Bruker MicroTOF instrument with an ESI source and Time of Flight (TOF) analyser was used. MS data are represented as a ratio of mass to

charge (m/z) in Daltons. A Bruker Tensor 27 instrument was used to obtain Fourier transform infrared spectra (FT-IR). Spectroscopic grade solvents and a Perkin Elmer 241 Polarimeter were used to obtain optical rotations.
All chemicals, reagents, and solvents were obtained from Sigma-Aldrich (Dorset, UK) and used without further purification. HPLC grade solvents were used for reactions, chromatography, and work-ups.

General Procedure for reductive amination (**General Procedure A**): The relevant amine (1 equiv) and aldehyde (1.2 equivs) were dissolved in either CH_2Cl_2 (4 ml). Sodium triacetoxyborohydride (5 equiv) and CH_3CO_2H (a few drops) were then added. The resultant reaction mixture was stirred at room temperature overnight. MeOH (5 ml) was then added and the resultant mixture was stirred for 5 mins. CH_2Cl_2 (15 ml) was then added and the reaction mixture was washed with water, brine and then dried over Na_2SO_4. The solvent was removed in *vacuo* and was purified by flash column chromatography using (0% - 5% MeOH, CH_2Cl_2, 1 % NH_3) over 20 column volumes to give the desired compound.

General Procedure for amide coupling (**General Procedure B**): The carboxylic acid (1equiv) and DIPEA (2.5 equiv) were dissolved in dimethylacetamide (DMAc) (5ml), followed by the addition of T3P (1.5 equiv, 50% in DMF). The resultant reaction mixture was stirred at room temperature for 30 mins before the addition of the amine (1.2 equiv). The resultant mixture was stirred overnight at room temperature. EtOAc (15 ml) was then added to the reaction mixture which was then washed with water, brine and dried with anhydrous Na_2SO_4. The crude compound was purified by flash column chromatography

using (Cyclohexane 100 % - 50%, EtOAc 0 %- 50 %) over 10 column volumes to give the desired compound.

tert-Butyl 2,4-dioxo-1,3,8-triazaspiro[4.5]decane-8-carboxylate 7

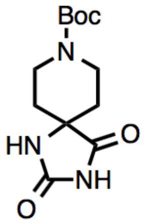

1-Butyloxycarbonyl-4-piperidone (**6**) (4g, 0.02 mol), potassium cyanide (1.95g, 0.03 mol), and ammonium carbonate (5.78 g, 0.06 mol) were suspended in water and EtOH (40 ml (1:1)). The resultant mixture was stirred at room temperature for 4 hr. The reaction mixture was then cooled, to enable precipitation. The precipitate was collected by filtration, and washed with ethanol to give **7** (3.55 g, 0.013 mol, 65.4 %) as a white crystalline solid.

m.p. >250°C. **IR** $_{\nu max}$ (film) 3427, 3342, 3050, 2910, 1729, 1713 cm^{-1}. ^{1}**H NMR** (400 MHz, DMSO-d_6) δ 10.72 (s, 1H, NH), 8.52 (s, 1H, NH), 3.86 – 3.74 (m, 2H, CH_2CH_2N), 3.22 – 3.01 (m, 2H, CH_2CH_2N), 1.73 – 1.62 (m, 2H, CH_2CH_2N), 1.56 – 1.47 (m, 2H,CH_2CH_2N), 1.40 (s, 9H, ^{t}Boc). ^{13}**C NMR** (101 MHz, DMSO-d_6) δ 177.96, 156.67, 154.29, 79.42, 60.60, 33.23, 28.49. **HRMS** (ESI-TOF) calcd for $C_{12}H_{19}O_4N_3{}^{23}Na_1$ [M+Na]$^+$: 292.1266, found : 292.1264.

tert-Butyl 3-([1,1'-biphenyl]-4-yl)-2,4-dioxo-1,3,8-triazaspiro[4.5]decane-8-carboxylate 8

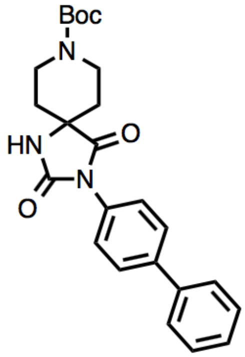

7 (500mg, 1.85 mmol) and 4-iodobiphenyl (518 mg, 1.85 mmol) were suspended in a mixture of CH_3CN and DMF (20 ml (1:1)). The mixture was degassed using a stream of N_2 for 15 min. *N,N'*-Dimethylethylenediamine (36.7 µl, 0.55 mmol), copper(I) iodide (104 mg, 0.55 mmol) and potassium carbonate (895 mg, 6.475 mmol) were then added sequentially. The resultant mixture was heated at 85°C for 16 hr, cooled to room temperature; then combined with EtOAc (40 ml) and water (40 ml). The organic layer was separated, washed with water (2 x 40 ml) and dried over Na_2SO_4. The volatiles were evaporated in *vacuo* and purified by flash column chromatography (cyclohexane 100 % - 50%, EtOAc 0 %- 50 %) over 10 column volumes to give **8** (340 mg, 0.805 mmol, 43 %) as a white solid.

m.p. 224-226°C. **IR** $_{\nu max}$ (film) 3342, 3050, 2910, 1719, 1702 cm^{-1}. **^{1}H NMR** (400 MHz, DMSO-*d*$_6$) δ 9.15 (s, 1H, NH), 7.82 – 7.68 (m, 4H, Ar), 7.57 – 7.37 (m, 5H, Ar), 3.90 (m, 2H, CH_2CH_2N), 3.39 – 3.34 (m, 2H, CH_2CH_2N), 1.92 – 1.80 (m, 2H, CH_2CH_2N), 1.82 – 1.67 (m, 2H, CH_2CH_2N), 1.44 (s, 9H, ^{t}Boc). **^{13}C NMR** (101 MHz, DMSO-*d*$_6$) δ 175.27, 154.98, 154.32, 140.05, 139.80, 131.76, 129.48, 128.21,

127.66, 127.39, 127.26, 79.51, 59.56, 33.30, 28.52. **HRMS** (ESI-TOF) calcd for $C_{24}H_{27}O_4N_3{}^{23}Na_1$ $[M+Na]^+$: 444.1893, found : 444.1891.

tert-Butyl 3-([1,1'-biphenyl]-4-yl)-2,4-dioxo-1-(pyrimidin-2-yl)-1,3,8-triazaspiro[4.5]decane-8-carboxylate 9

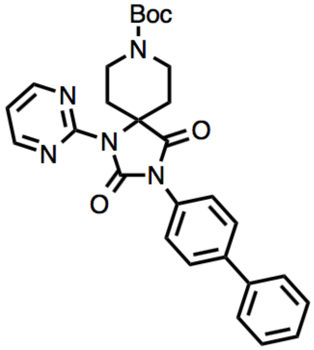

A solution of **8** (400 mg, 0.94 mmol). 2-iodo-pyrimidine (585 mg, 2.84 mmol) in a mixture of CH_3CN and DMF (20 ml (1:1)) was degassed with a stream of N_2 for 15 min. 2,2,6,6-Tetramethyl-3,5-heptadion (196 µl, 0.94 mmol), copper(I) iodide (179 mg, 0.94 mmol) and Cs_2CO_3 (1.5 g, 4.475 mmol) were then added sequentially. The resultant mixture was heated at 85℃ for 16 hr, cooled to room temperature then combined with EtOAc (40 ml) and water (40 ml). The organic layer was separated, washed with water (2 x 40 ml), and dried over Na_2SO_4. The volatiles were evaporated in *vacuo* and purified by flash column chromatography (cyclohexane 100% - 75%, EtOAc 0% - 25%) over 10 column volumes gave **9** (280 mg, 0.56 mmol, 60 %) as a white solid.

m.p. 220-223℃. **IR** $_{\nu max}$ (film) 2936, 1766, 1675, 1517 cm^{-1}. **^{1}H NMR** (400 MHz, DMSO-d_6) δ 8.82 (d, J = 5.0 Hz, 2H, pyrimidine-H), 7.86 – 7.38 (m, 10H, Ar), 4.04 – 3.88 (m, 2H, CH_2CH_2N), 3.57 – 3.37 (m, 2H, CH_2CH_2N), 2.71 – 2.57 (m, 2H,

CH_2CH_2N), 2.18 – 2.11 (m, 2H, CH_2CH_2N), 1.43 (s, 9H, ^{t}Boc). **^{13}C NMR** (101 MHz, DMSO-d_6) δ 172.98, 158.62, 155.79, 154.22, 151.58, 140.30, 139.26, 130.63, 129.03, 128.00, 127.11, 126.88, 118.86, 78.99, 63.12, 39.52, 28.06. **HRMS** (ESI-TOF) calcd for $C_{28}H_{30}O_4N_5$ $[M+H]^+$: 500.2292, found: 500.2291.

3-([1,1'-Biphenyl]-4-yl)-1-(pyrimidin-2-yl)-1,3,8-triazaspiro[4.5]decane-2,4-dione 10

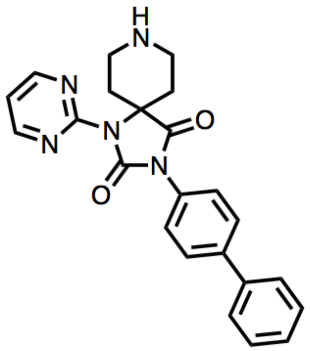

9 (250 mg, 0.501 mmol) was dissolved in neat formic acid (10ml) and stirred at room temperature for 3 hr. The volatiles were evaporated in *vacuo* to give **10** (199 mg, apparent quant) as a orange solid.

m.p. 181-183℃. **IR** $_{\nu max}$ (film) 3347, 2932, 1683, 1541 cm^{-1}. **^{1}H NMR** (400 MHz, DMSO-d_6) δ 8.88 (d, J = 5.0 Hz, 2H, pyrimidine-H), 8.02 – 7.23 (m, 10H, Ar), 3.47 – 3.28 (m, 4H, CH_2CH_2N), 2.83 (m, 2H, CH_2CH_2N), 2.39 – 2.20 (m, 2H, CH_2CH_2N). **^{13}C NMR** (101 MHz, DMSO-d_6) δ 173.08, 164.87, 159.30, 155.98, 152.12, 140.81, 139.69, 130.99, 129.50, 128.42, 128.34, 127.56, 127.33, 119.77, 62.11, 40.38, 38.14, 28.28. **HRMS** (ESI-TOF) calcd for $C_{23}H_{22}O_2N_5$ $[M+H]^+$: 400.1768, found : 400.1763.

3-([1,1'-Biphenyl]-4-yl)-8-((3-methylpyridin-2-yl)methyl)-1-(pyrimidin-2-yl)-1,3,8-triazaspiro[4.5]decane-2,4-dione 11

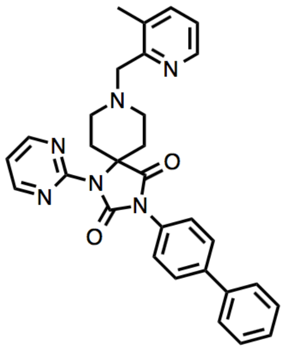

Following general procedure A: **10** (30 mg, 0.075 mmol), 3-methylpyridine-2-carboxaldehyde (10.12 µl, 0.090 mmol) and sodium triacetoxyborohydride (72 mg, 0.350 mmol) gave **11** (13 mg, 0.025 mmol, 35 %) as a white solid.

m.p. 223-226℃. **IR** $_{\nu max}$ (film) 2980, 1671, 1545 cm^{-1}. **^{1}H NMR** (400 MHz, DMSO-*d*$_6$) δ 8.87 (d, J = 5.0 Hz, 2H, pyrimidine-H), 8.30 (m, 1H), 7.88 – 7.36 (m, 11H, Ar), 7.19 (m, 1H), 3.66 (s, 2H, Ar-CH_2), 3.00 – 2.57 (m, 6H, CH_2CH_2N), 2.38 (s, 3H, CH_3), 2.15 – 2.00 (m, 2H, CH_2CH_2N). **^{13}C NMR** (101 MHz, DMSO-*d*$_6$) δ 173.57, 172.46, 159.26, 156.31, 152.48, 146.20, 140.66, 139.74, 138.29, 133.33, 131.21, 129.49, 128.37, 127.56, 127.32, 122.90, 119.72, 63.90, 48.89, 30.67, 21.53, 18.48. **HRMS** (ESI-TOF) calcd for $C_{30}H_{29}O_2N_6$ [M+H]$^+$: 505.2347, found : 505.2342.

3-([1,1'-Biphenyl]-4-yl)-1-(pyrimidin-2-yl)-8-(thiophen-2-ylmethyl)-1,3,8-triazaspiro[4.5]-decane-2,4-dione 12

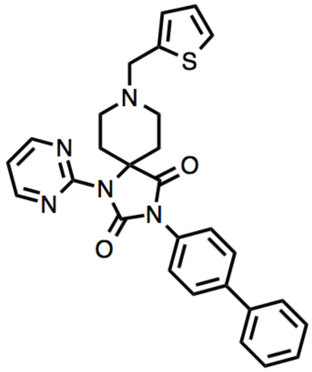

Following general procedure B: **10** (15 mg, 0.037 mmol), 2-thiophene carboxaldehyde (8.4 mg, 0.0751 mmol) and sodium triacetoxyborohydride (39 mg, 0.185 mmol) gave **12** (16 mg, 0.032 mmol, 84 %) as a cream solid.

m.p. 207-209°C. **IR** $_{\nu max}$ (film) 2982, 1699, 1644, 1554 cm^{-1}. **^{1}H NMR** (400 MHz, DMSO-d_6) δ 8.90 (d, J = 5.00 Hz, 2H, pyrimidine-H), 7.92 – 7.31 (m, 10H, Ar), 6.99 – 6.94 (m, 2H, thiophene-H), 3.77 (s, 2H, Ar-CH_2), 2.83 – 2.64 (m, 6H, CH_2CH_2N), 2.10 (m, 2H, CH_2CH_2N). **^{13}C NMR** (101 MHz, DMSO-d_6) δ 173.07, 158.82, 155.85, 152.00, 141.69, 140.19, 139.26, 130.73, 129.03, 127.93, 127.09, 126.86, 126.54, 126.18, 125.41, 119.24, 63.39, 55.74, 47.83, 39.52, 30.26, 21.08. **HRMS** (ESI-TOF) calcd for $C_{28}H_{26}O_2N_5{}^{32}S$ [M+H]$^+$: 496.1802, found : 496.1799.

3-([1,1'-Biphenyl]-4-yl)-8-((1-methyl-1H-imidazol-2-yl)methyl)-1-(pyrimidin-2-yl)-1,3,8-triazaspiro[4.5]decane-2,4-dione 13

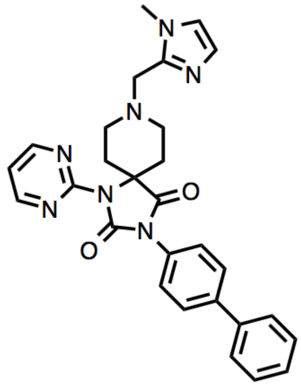

Following general procedure B: **10** (30 mg, 0.075 mmol), 1-methyl-2-imidazole carboxaldehyde (9.9 mg, 0.090 mmol) and sodium triacetoxyborohydride (72 mg, 0.350 mmol) gave **13** (18 mg, 0.036 mmol, 53 %) as a cream solid.

m.p. 228-230°C. **IR** ν_{max} (film) 2870, 1716, 1560 cm^{-1}. **^{1}H NMR** (400 MHz, DMSO-d_6)

δ 8.88 (d, J = 5.0 Hz, 2H, pyrimidine-H), 8.11 – 7.29 (m, 10H, Ar), 7.07 (s, 1H), 6.75 (s, 1H), 3.65 (s, 3H, CH_3), 3.59 (s, 2H, Ar-CH_2), 2.86 – 2.61 (m, 6H, CH_2CH_2N), 2.10 – 2.05 (m, 2H, CH_2CH_2N). **^{13}C NMR** (101 MHz, DMSO-d_6) δ 178.31, 164.05, 161.07, 157.18, 145.42, 144.49, 135.96, 134.24, 133.14, 133.05, 132.31, 132.08, 131.36, 127.09, 124.41, 68.56, 58.15, 53.36, 37.71, 35.27. **HRMS** (ESI-TOF) calcd for $C_{28}H_{28}O_2N_7$ $[M+H]^+$: 494.2299, found : 494.2289.

3-([1,1'-Biphenyl]-4-yl)-8-(2-hydroxybenzyl)-1-(pyrimidin-2-yl)-1,3,8-triazaspiro[4.5]decane-2,4-dione 14

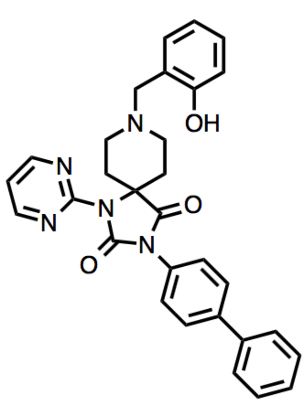

Following general procedure B: **10** (30 mg, 0.075 mmol), salicaldehyde (9.5 µl, 0.090 mmol) and sodium triacetoxyborohydride (72 mg, 0.350 mmol) gave **14** (15 mg, 0.030 mmol, 40 %) as a cream solid.

m.p. 225-229℃. **IR** ν_{max} (film) 3387, 2980, 1717 cm^{-1}. **^{1}H NMR** (400 MHz, DMSO-d_6) δ 8.90 (d, J = 5.0 Hz, 2H, pyrimidine-H), 7.86 – 6.71 (m, 15H, Ar), 3.72 (s, 2H, Ar-CH_2), 2.89 – 2.82 (m, 4H, CH_2CH_2N), 2.84 – 2.70 (m, 2H, CH_2CH_2N), 2.21 – 2.13 (m, 2H, CH_2CH_2N). **^{13}C NMR** (101 MHz, DMSO-d_6) δ 173.50, 159.23, 157.36, 156.28, 152.31, 140.70, 139.72, 131.17, 129.79, 129.49, 128.61, 128.42, 128.30, 127.56, 127.33, 122.87, 119.59, 119.24, 115.77, 63.52, 58.62, 48.47, 30.49. **HRMS** (ESI-TOF) calcd for $C_{30}H_{26}O_3N_5$ $[M-H]^-$: 504.2041, found : 504.2049.

8-((1H-Imidazol-4-yl)methyl)-3-([1,1’-biphenyl]-4-yl)-1-(pyrimidin-2-yl)-1,3,8-triazaspiro[4.5]decane-2,4-dione 15

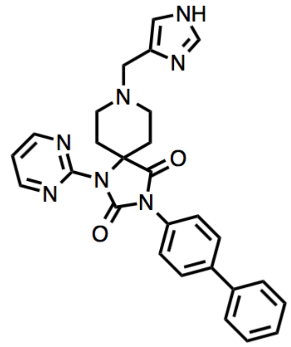

Following general procedure B: **10** (30 mg, 0.075 mmol), 4-imidazole-carboxaldehyde (10.7 mg, 0.090 mmol) and sodium triacetoxyborohydride (72 mg, 0.350 mmol) gave **15** (17 mg, 0.035 mmol, 48 %) as a brown solid.

m.p. 138-143℃. **IR** ν_{max} (film) 3480, 2906, 1776, 1713, 1563 cm^{-1}. **^{1}H NMR** (400 MHz, DMSO-d_6) δ 8.88 (d, J = 5.00 Hz, 2H, pyrimidine-H), 7.90 – 7.33 (m, 12H, Ar),

3.47 (s, 2H, Ar-CH_2), 2.82 – 2.61 (m, 6H, CH_2CH_2N), 2.14 – 2.02 (m, 2H, CH_2CH_2N). **^{13}C NMR** (101 MHz, DMSO-d_6) δ 173.07, 158.82, 155.85, 152.00, 141.69, 140.19, 139.26, 130.73, 129.03, 127.93, 127.09, 126.86, 126.54, 126.18, 125.41, 119.24, 63.39, 55.74, 47.83, 39.52, 30.26, 21.08. **HRMS** (ESI-TOF) calcd for $C_{27}H_{26}O_2N_7$ $[M+H]^+$: 480.2142, found : 480.2140.

3-([1,1’-Biphenyl]-4-yl)-8-((6-methoxypyridin-2-yl)methyl)-1-(pyrimidin-2-yl)-1,3,8-triazaspiro[4.5]decane-2,4-dione 16

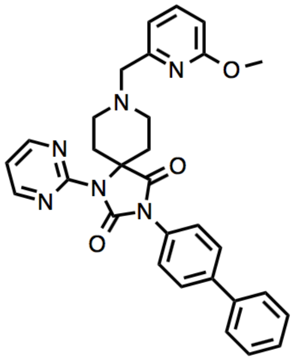

Following general procedure B: **10** (30 mg, 0.075 mmol), 1-methyl-2-imidazole carboxaldehyde (13.47 µl, 0.112 mmol) and sodium triacetoxyborohydride (72 mg, 0.350 mmol) gave **16** (14 mg, 0.027 mmol, 35 %) as a white solid.

m.p. 210-212℃. **IR** $_{νmax}$ (film) 2902, 1751, 1636, 1457 cm^{-1}. **^{1}H NMR** (400 MHz, DMSO-d_6) δ 8.91 (d, J = 5.0 Hz, 2H, pyrimidine-H), 7.85 – 7.37 (m, 11H, Ar), 7.02 (d, J = 7.0 Hz, 1H), 6.68 (d, J = 8.0 Hz, 1H), 3.83 (s, 3H, O-CH_3), 3.61 (s, 2H, Ar-CH_2), 2.93 – 2.67 (m, 5H), 2.10 (d, J = 12.5 Hz, 2H), 1.91 (s, 1H). **^{13}C NMR** (101 MHz, DMSO-d_6) δ 173.57, 163.42, 159.28, 156.75, 156.34, 152.48, 140.66, 139.80, 139.73, 131.22, 129.49, 128.39, 127.56, 127.32, 119.71, 115.91, 108.87, 63.84,

63.35, 53.34, 48.76, 30.90. **HRMS** (ESI-TOF) calcd for $C_{30}H_{29}O_3N_6$ $[M+H]^+$: 521.2296, found : 521.2293.

tert-Butyl 3-([1,1'-biphenyl]-4-yl)-1-(4-(methoxycarbonyl)pyridin-2-yl)-2,4-dioxo-1,3,8-triazaspiro[4.5]decane-8-carboxylate 18

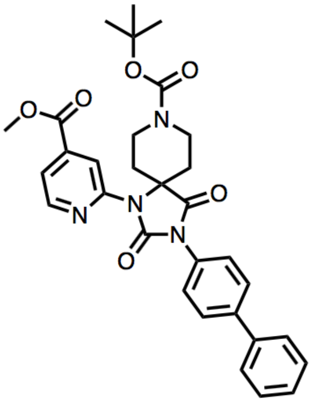

A solution **8** (50mg, 0.118 mmol) and methyl 2-iodoisonicotinate (93 mg, 0.356 mmol) was suspended in a mixture of CH_3CN and DMF (5 ml (1:1)). The mixture was degassed using stream of N_2 for 15 min. 2,2,6,6-Tetramethyl-3,5-heptadion (25 µl, 0.118 mmol), copper (I) iodide (22 mg, 0.118 mmol) and Cs_2CO_3 (192 mg, 0.59 mmol) were then added sequentially. The resultant mixture was heated at 85℃ for 48 hrs, cooled to room temperature and combined with EtOAc (15 ml) and water (15 ml). The organic layer was separated, washed with water (2 x 40 ml) and dried with anhydrous Na_2SO_4. The volatiles were evaporated in *vacuo* and purified by flash column chromatography using (cyclohexane 100 % - 50%, EtOAc 0 % - 50 %) over 10 column volumes to give **18** (29 mg, 0.052 mmol, 45 %) as an clear oil.

IR ν_{max} (film) 2921, 1742, 1730, 1662, 1517 cm^{-1}. **^{1}H NMR** (400 MHz, chloroform-*d*) δ 8.66 (d, J = 1.0 Hz, 1H, pyridine-H), 8.50 (dd, J = 5.0, 1.0 Hz, 1H, pyridine-H), 7.79 – 7.35 (m, 10H, Ar), 4.33 – 4.03 (m, 2H, CH_2CH_2N), 3.94 (s, 3H, CH_3), 3.59 (m, 2H,

CH_2CH_2N), 3.18 (m, 2H, CH_2CH_2N), 1.88 (m, 2H, CH_2CH_2N), 1.52 (s, 9H, ^{t}Boc). **^{13}C NMR** (101 MHz, Chloroform-*d*) δ 173.56, 165.22, 155.13, 153.36, 151.00, 148.27, 141.80, 140.22, 139.75, 130.03, 129.00, 128.04, 127.88, 127.36, 126.78, 119.69, 117.67, 79.91, 77.16, 64.36, 52.95, 39.49, 28.58, 27.02. **HRMS** (ESI-TOF) calcd for $C_{31}H_{33}O_6N_4$ $[M+H]^+$: 557.2395, found : 557.2289.

tert-Butyl 3-([1,1'-biphenyl]-4-yl)-1-(4-methoxypyrimidin-2-yl)-2,4-dioxo-1,3,8-triazaspiro[4.5]decane-8-carboxylate 19

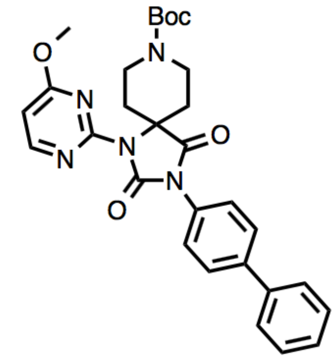

A solution of **8** (400 mg, 0.94 mmol) and 2-iodo-4-methoxypyrimidine (670 mg, 2.84 mmol) was suspended in a mixture of CH_3CN and DMF (20 ml (1:1)). The mixture degassed using stream of N_2 for 15 min. 2,2,6,6-Tetramethyl-3,5-heptadion (196 µl, 0.94 mmol), copper (I) iodide (179 mg, 0.94 mmol) and Cs_2CO_3 (1.5 g, 4.475 mmol) were then added sequentially. The resultant mixture was heated at 85℃ for 48 hrs, cooled to room temperature and combined with EtOAc (40 ml) and water (40 ml). The organic layer was separated, washed with water (2 x 40 ml) and dried over Na_2SO_4. The volatiles were evaporated in *vacuo* and purified by flash column chromatography using (cyclohexane 100 % - 75%, EtOAc 0 %- 25 %) over 10 column volumes to give **19** (342 mg, 0.64 mmol, 69 %) as clear oil.

IR ν_{max} (film) 2921, 1730, 1662, 1517 cm^{-1}. **^{1}H NMR** (400 MHz, chloroform-*d*) δ 8.32 (d, J = 5.5 Hz, 1H, pyrimidine-H), 7.66 – 7.26 (m, 9H, Ar), 6.50 (d, J = 5.5 Hz, 1H, pyrimidine-H), 4.21 – 4.16 (m, 2H, CH_2CH_2N), 4.10 – 4.00 (m, 2H, CH_2CH_2N), 3.90 (s, 3H, O-CH_3), 3.61 – 3.40 (m, 2H, CH_2CH_2N), 3.15 – 2.92 (m, 2H, CH_2CH_2N), 1.42 (s, 3H, ^{t}Boc), 1.36 (s, 6H, ^{t}Boc). **^{13}C NMR** (101 MHz, Chloroform-*d*) δ 173.32, 169.94, 157.88, 155.66, 154.64, 151.84, 141.63, 140.20, 130.02, 128.88, 127.90, 127.27, 126.81, 104.99, 79.82, 63.71, 54.47, 28.46, 26.94. **HRMS** (ESI-TOF) calcd for $C_{29}H_{32}O_5N_5$ $[M+H]^+$: 530.2398, found : 530.2397.

3-([1,1'-Biphenyl]-4-yl)-1,3,8-triazaspiro[4.5]decane-2,4-dione 20

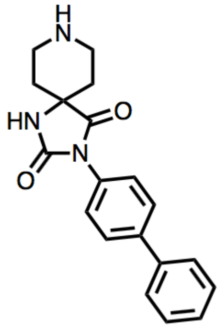

8 (330 mg, 0.652 mmol) was dissolved in CH_2Cl_2 (5 ml) and HCl ((2M) in ether (3 ml)) was added to the solution. The resultant mixture was put under *vacuo* and then flushed with N_2, this was repeated 3 times. The resultant mixture was stirred at room temperature for 16 hrs. The volatiles were evaporated in *vacuo* to give **20** (330 mg, apparent quant) with the appearance of a clear oil.

IR ν_{max} (film) 3532, 3385, 2901, 1729, 1631, 1504 cm^{-1}. **^{1}H NMR** (400 MHz, DMSO-d_6) δ 9.26 (s, 1H, Amide NH), 7.82 – 7.35 (m, 9H, Ar), 3.48 – 3.29 (m, 2H, CH_2CH_2N), 3.26 – 3.04 (m, 2H, CH_2CH_2N), 2.30 – 2.11 (m, 2H, CH_2CH_2N), 2.11 – 1.95 (m, 2H, CH_2CH_2N). **^{13}C NMR** (101 MHz, DMSO-d_6) δ 174.44, 164.71, 154.61, 139.86,

139.41, 131.26, 129.18, 127.93, 127.40, 127.07, 126.92, 57.55, 39.52, 39.10, 30.16. **HRMS** (ESI-TOF) calcd for $C_{19}H_{20}O_2N_3$ $[M+H]^+$: 322.1550, found : 322.1544.

Methyl 2-(3-([1,1'-biphenyl]-4-yl)-2,4-dioxo-1,3,8-triazaspiro[4.5]decan-1-yl)isonicotinate 21

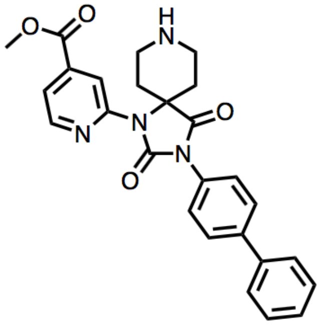

18 (29 mg, 0.0521 mmol) was dissolved in CH_2Cl_2 (5 ml) and HCl (2M) in ether (3 ml) was added to the solution. The resultant mixture was put under *vacuo* and flushed with N_2; this was repeated 3 times. The resultant mixture was stirred at room temperature for 16 hrs. The volatiles were evaporated in *vacuo* to give **21** (20 mg, apparent quantitative) as an orange oil.

IR ν_{max} (film) 3360, 2921, 1746, 1662, 1517 cm^{-1}. **^{1}H NMR** (400 MHz, DMSO-d_6) δ 9.06 – 8.94 (m, 1H, NH), 8.70 (d, J = 5.0 Hz, 1H, pyridine-H), 8.43 (d, J = 1.0 Hz, 1H, pyridine-H), 7.93 – 7.37 (m, 10H, Ar), 3.91 (s, 3H, CH_3), 3.49 – 3.33 (m, 4H, CH_2CH_2N), 3.10 – 2.99 (m, 2H, CH_2CH_2N), 2.47 – 2.38 (m, 2H, CH_2CH_2N). **^{13}C NMR** (101 MHz, DMSO-d_6) δ 173.03, 165.03, 153.43, 150.75, 149.51, 140.68, 139.52, 139.36, 130.76, 129.40, 128.23, 127.37, 127.20, 120.20, 118.60, 61.85, 53.36, 39.52, 27.40. **HRMS** (ESI-TOF) calcd for $C_{26}H_{25}O_4N_4$ $[M+H]^+$: 457.1870, found : 457.1862.

3-([1,1'-Biphenyl]-4-yl)-1-(4-methoxypyrimidin-2-yl)-1,3,8-triazaspiro[4.5]decane-2,4-dione 22

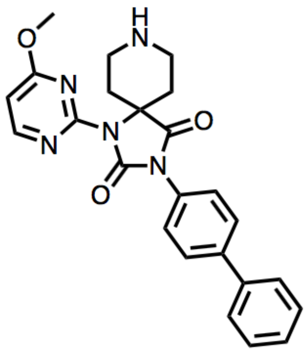

19 (345 mg, 0.652mmol) was dissolved in neat formic acid (10 ml) and stirred at room temperature for 3 hrs. The volatiles were evaporated in *vacuo* to give **22** (240 mg, 0.55 mmol, 86 %) as a yellow oil.

IR ν_{max} (film) 3342 ,2929, 1720, 1675, 1550 cm^{-1}. **^{1}H NMR** (400 MHz, DMSO-d_6) δ 8.54 (d, J = 6.00 Hz, 1H, pyrimidine-H), 7.78 – 7.37 (m, 9H), 6.89 (d, J = 6.00 Hz, 1H, pyrimidine-H), 4.00 (s, 3H, O-CH_3), 3.39 (m, 2H, CH_2CH_2N), 3.28 (m, 2H, CH_2CH_2N), 2.89 (m, 2H, CH_2CH_2N), 2.33 (m, 2H, CH_2CH_2N). **^{13}C NMR** (101 MHz, DMSO-d_6) δ 173.08, 170.31, 158.92, 151.92, 140.80, 139.72, 131.03, 129.78, 129.50, 128.43, 128.33, 127.66, 127.54, 127.33, 105.87, 62.39, 54.88, 28.77. **HRMS** (ESI-TOF) calcd for $C_{24}H_{24}O_3N_5$ [M+H]$^+$: 430.1873, found : 430.1867.

3-([1,1'-Biphenyl]-4-yl)-8-((3-methylpyridin-2-yl)methyl)-1,3,8-triazaspiro[4.5]-decane-2,4-dione 23

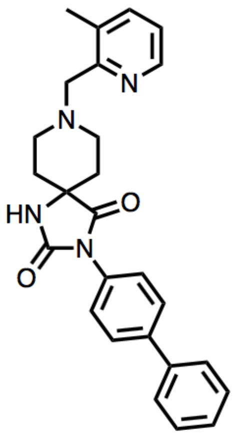

Following general procedure A: **20** (280 mg, 0.87 mmol), 3-methylpyridine-2-carboxaldehyde (113 µl, 1.74 mmol) and sodium triacetoxyborohydride (985 mg, 4.65 mmol) gave **23** (180 mg, 0.422 mmol, 49 %) as an orange oil.

IR ν_{max} (film) 3556, 1720, 1644, 1504 cm^{-1}. **^{1}H NMR** (400 MHz, DMSO-d_6) δ 9.10 (s, 1H, NH), 8.30 (dd, J = 4.5, 1.5 Hz, 1H, pyridine-H), 7.81 – 7.16 (m, 11H, Ar), 3.64 (s, 2H, CH_2), 2.80 – 2.70 (m, 2H, CH_2CH_2N), 2.48 – 2.42 (m, 2H, CH_2CH_2N), 2.40 (s, 3H, CH_3), 2.03 – 1.80 (m, 2H, CH_2CH_2N), 1.78 – 1.62 (m, 2H, CH_2CH_2N). **^{13}C NMR** (126 MHz, DMSO-d_6) δ 175.48, 156.35, 154.56, 145.70, 139.56, 139.37, 137.93, 133.12, 131.43, 129.06, 127.77, 127.23, 126.81, 122.59, 62.63, 59.23, 48.62, 48.31, 39.52, 33.32, 29.05, 17.95. **HRMS** (ESI-TOF) calcd for $C_{26}H_{27}O_2N_4$ $[M+H]^+$: 427.2129, found 427.2127.

3-([1,1’-Biphenyl]-4-yl)-8-((1-methyl-1*H*-imidazol-2-yl)methyl)-1,3,8-triazaspiro[4.5]-decane-2,4-dione 24

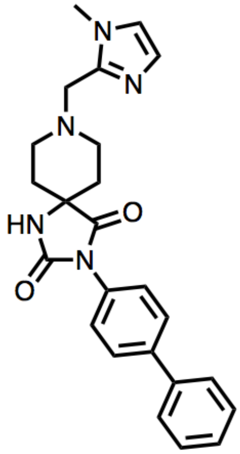

Following general procedure A: **20** (280 mg, 0.87 mmol), 1-methyl–2-imidazole-carboxaldehyde (113 µl, 1.74 mmol) and sodium triacetoxyborohydride (985 mg, 4.65 mmol) gave **24** (321 mg, 0.77 mmol, 89 %) as a white solid.

m.p. >250℃. **IR** ν_{max} (film) 3556, 1724, 1641, 1504 cm^{-1}. **^{1}H NMR** (400 MHz, DMSO-*d*$_6$) δ 9.06 (s, 1H, NH), 7.80 – 7.67 (m, 4H, Ph), 7.54 – 7.43 (m, 4H, Ph), 7.45 – 7.35 (m, 1H, Ph), 7.10 (d, *J* = 1.0 Hz, 1H, imidazole-H), 6.77 (d, *J* = 1.0 Hz, 1H, imidazole-H), 3.67 (s, 3H, CH_3), 3.58 (s, 2H, $\underline{CH_2}$imidazole), 2.79 – 2.69 (m, 2H, CH_2CH_2N), 2.45 – 2.34 (m, 2H, CH_2CH_2N), 1.99 – 1.87 (m, 2H, CH_2CH_2N), 1.78 – 1.70 (m, 2H, CH_2CH_2N). **^{13}C NMR** (101 MHz, DMSO) δ 175.86, 154.99, 144.72, 139.99, 139.81, 131.87, 129.47, 128.18, 127.64, 127.37, 127.24, 126.60, 122.52, 59.62, 54.38, 48.47, 33.69, 32.94. **HRMS** (ESI-TOF) calcd for $C_{24}H_{26}O_2N_5$ [M+H]$^+$: 416.2081, found : 416.2079.

Methyl 2-(3-([1,1'-biphenyl]-4-yl)-8-((3-methylpyridin-2-yl)methyl)-2,4-dioxo-1,3,8-triazaspiro[4.5]decan-1-yl)isonicotinate 25

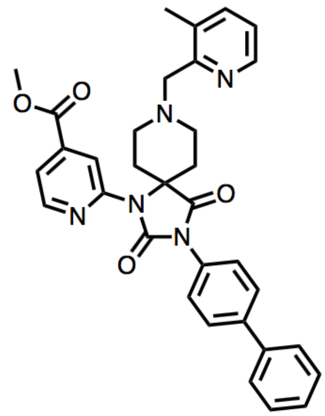

Following general procedure A: **21** (20 mg, 0.0438 mmol), 3-methylpyridine-2-carboxaldehyde (6 µl,, 0.0625 mmol) and sodium triacetoxyborohydride (46 mg, 0.219 mmol) gave **25** (14 mg, 0.0249 mmol, 57 %) as a clear oil.

IR ν_{max} (film) 2921, 1744, 1662, 1517 cm^{-1}. **^{1}H NMR** (400 MHz, THF-*d*$_8$) δ 8.61 (t, *J* = 1.0 Hz, 1H, pyridine-H), 8.57 (dd, *J* = 5.0, 1.0 Hz, 1H, pyridine-H), 8.26 (dd, *J* =

5.0, 1.0 Hz, 1H, pyridine-H), 7.79 – 7.30 (m, 12H, Ar), 3.90 (s, 3H, CH_3), 3.77 (s, 2H, CH_2-Pyr), 3.26 – 3.01 (m, 4H, CH_2CH_2N), 2.87 – 2.74 (m, 2H, CH_2CH_2N), 2.48 (s, 3H, O-CH_3), 1.94 – 1.81 (m, 2H, CH_2CH_2N). **^{13}C NMR** (101 MHz, THF-d_8) δ 174.16, 165.78, 158.36, 154.17, 152.85, 149.26, 146.87, 146.24, 140.48, 138.41, 134.32, 132.37, 129.77, 128.48, 128.00, 127.83, 123.09, 119.99, 119.20, 67.57, 65.21, 63.67, 53.02, 49.82, 31.18, 18.74. **HRMS** (ESI-TOF) calcd for $C_{33}H_{32}O_4N_5$ $[M+H]^+$: 562.2448, found : 562.2437.

3-([1,1'-Biphenyl]-4-yl)-1-(4-methoxypyrimidin-2-yl)-8-((3-methylpyridin-2-yl)methyl)-1,3,8-triazaspiro[4.5]decane-2,4-dione 26

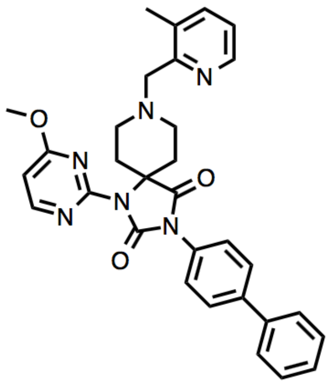

Following general procedure A: **22** (46 mg, 0.107 mmol), 3-methylpyridine-2-carboxaldehyde (14.35 µl, 0.128 mmol) and sodium triacetoxyborohydride (113 mg, 0.535 mmol) gave **26** (18 mg, 0.033 mmol, 32 %) as a cream solid.

m.p. 173-177°C. **IR** $_{νmax}$ (film) 2980, 1740, 1653, 1572 cm^{-1}. **^{1}H NMR** (400 MHz, DMSO-d_6) δ 8.44 (d, J = 6.00 Hz, 1H, pyrimidine-H), 8.22 (dd, J = 5.00, 2.00 Hz, 1H), 7.85 – 7.06 (m, 11H, Ar), 6.74 (d, J = 6.00 Hz, 1H, pyrimidine-H), 3.86 (m, 2H, CH_2), 3.59 (s, 3H, O-CH_3), 2.90 – 2.62 (m, 4H, CH_2CH_2N), 2.45 – 2.40 (m, 2H,

CH_2CH_2N), 2.32 (s, 3H, Pyridine-CH_3), 1.96 (m, 2H, CH_2CH_2N). **^{13}C NMR** (101 MHz, DMSO-d_6) δ 173.49, 169.93, 158.98, 156.95, 155.80, 151.94, 146.15, 140.68, 139.75, 138.25, 133.40, 131.20, 129.49, 128.43, 128.29, 127.54, 127.32, 122.94, 105.36, 63.88, 62.53, 54.60, 49.11, 30.21, 18.25. **HRMS** (ESI-TOF) calcd for $C_{31}H_{31}O_3N_6$ $[M+H]^+$: 535.2452, found : 535.2449.

2-(3-([1,1'-Biphenyl]-4-yl)-8-((3-methylpyridin-2-yl)methyl)-2,4-dioxo-1,3,8-triazaspiro[4.5]-decan-1-yl)isonicotinic acid 27

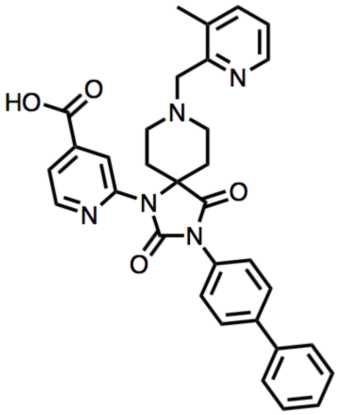

25 (12 mg, 0.024 mmol) was dissolved in a mixture of THF and water (10 :1 (3 ml)) before the addition of LiOH-H_2O (2 mg, 0.048 mmol). The reaction mixture were stirred for 16 hrs before neutralized with HCl_{aq} (1M). The THF was removed in *vacuo* and the resulting aqueous solution was purified using prep HPLC. The fractions were combined and the solvent removed in *vacuo* to give **27** (6 mg, 0.0109 mmol, 46 %) as a white solid.

IR ν_{max} (film) 2921, 2705, 1763, 1662, 1517 cm^{-1}. **^{1}H NMR** (400 MHz, DMSO-d_6) δ 8.84 – 8.33 (m, 2H, Ar), 8.07 – 7.31 (m, 13H, Ar), 4.69 (s, 2H, CH_2), 3.22 (m, 4H,

CH_2CH_2N), 2.55 (m, 4H, CH_2CH_2N), 2.35 (s, 3H, CH_3). ^{13}C NMR (126 MHz, DMSO-d_6) δ 172.70, 165.66, 158.33, 158.07, 157.80, 157.54, 153.07, 150.58, 150.31, 149.13, 148.96, 146.18, 140.63, 140.28, 139.17, 138.79, 135.99, 132.02, 130.48, 129.06, 127.91, 127.78, 127.01, 126.86, 123.56, 120.36, 119.83, 115.40, 60.86, 60.18, 48.73, 39.52, 24.26, 18.53, 17.20. **HRMS** (ESI-TOF) calcd for $C_{32}H_{30}O_4N_5$ $[M+H]^+$: 548.2292, found : 548.2286.

Benzyl 4-([1,1'-biphenyl]-4-ylcarbamoyl)piperidine-1-carboxylate 29

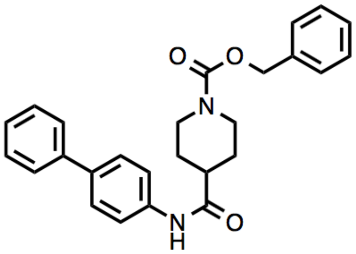

Following general procedure B: 1-Benzyloxycarbonylpiperidine-4-carboxylic acid (**28**) (1.43 g, 5.46 mmol) and 4-amino-biphenyl (500mg, 2.73 mmol) gave **29** (905 mg, 2.18 mmol, 83%) as a white solid.

m.p. 179 - 181 °C. **IR** $_{\nu max}$ (film) 3280, 1699, 1651. cm^{-1}. **^{1}H NMR** (400 MHz, DMSO-d_6) δ 10.03 (s, 1H, NH), 7.76 – 7.58 (m, 6H, Ar), 7.52 – 7.26 (m, 8H, Ar), 5.09 (s, 2H, O-$\underline{CH_2}$), 2.89 (m, 2H, CH_2CH_2N), 2.57 (m, 1H, CH_2CH_2N), 1.89 – 1.46 (m, 6H, CH_2CH_2N). **^{13}C NMR** (101 MHz, DMSO-d_6) δ 172.96, 154.40, 139.70, 138.75, 137.01, 134.71, 128.88, 128.43, 127.83, 127.54, 126.97, 126.84, 126.20, 119.47, 66.17, 43.05, 42.51, 28.20. **HRMS** (ESI-TOF) calcd for $C_{26}H_{25}N_2O_3$ $[M-H]^-$: 413.1871, found 413.1872.

Benzyl 4-(([1,1'-biphenyl]-4-ylmethyl)carbamoyl)piperidine-1-carboxylate 30

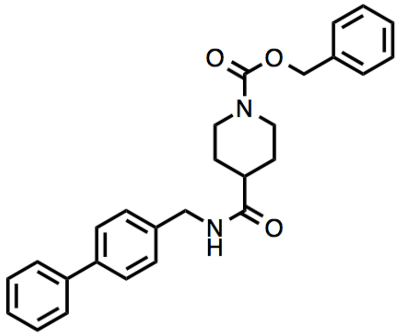

Following general procedure B: 1-Benzyloxycarbonylpiperidine-4-carboxylic acid (**28**) (431mg, 1.63 mmol) and 4-phenylbenzylamine (250mg, 1.36 mmol) gave **30** (392 mg, 0.915 mmol, 56 %) as a white solid.

m.p. 144 -145 ℃. **IR** ν_{max} (film) 3060, 1696, 1630 cm^{-1}. **^{1}H NMR** (400 MHz, chloroform-*d*) δ 7.72 – 7.14 (m, 14H, Ar), 5.12 (s, 2H, O-CH_2), 4.47 (d, J = 5.5 Hz, 2H, CH_2-NH), 4.23 (m, 2H, CH_2CH_2N), 2.83 (m, 2H, CH_2CH_2N), 2.45 – 2.17 (m, 1H, CH_2CH_2N), 1.94 – 1.63 (m, 4H, CH_2CH_2N). **^{13}C NMR** (101 MHz, Chloroform-*d*) δ 174.11, 155.28, 140.71, 137.29, 136.82, 128.94, 128.62, 128.32, 128.14, 128.01, 127.61, 127.53, 127.16, 77.16, 67.28, 43.57, 43.36, 43.30, 28.72. **HRMS** (ESI-TOF) calcd for $C_{27}H_{28}N_2O_3$ $[M+H]^+$: 429.2173, found 429.2183.

Benzyl 3-([1,1'-biphenyl]-4-ylcarbamoyl)piperidine-1-carboxylate 32

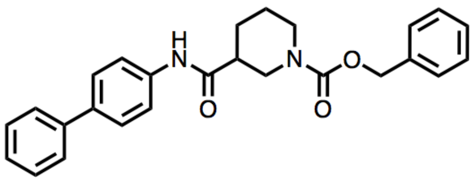

Following general procedure B: 1-Benzyloxycarbonylpiperidine-3-carboxylic acid (**31**) (300 mg, 1.14 mmol) and 4-amino-biphenyl (229 mg, 1.36 mmol) gave **32** (116 mg, 0.279 mmol, 25 %) as a white solid.

m.p. 144-148°C. **IR** $_{νmax}$ (film) 3034, 1696, 1653 cm^{-1}. **^{1}H NMR** (400 MHz, chloroform-*d*) δ 7.66 – 7.28 (m, 14H, Ar), 5.25 – 5.08 (s, 2H, CH_2), 4.39 – 3.80 (m, 2H, CH_2CH_2N), 3.51 – 2.84 (m, 2H, CH_2CH_2N), 2.58 – 2.37 (m, 1H, CH_2CH_2N), 2.19 – 1.93 (m, 1H, CH_2CH_2N), 1.84 – 1.43 (m, 3H, CH_2CH_2N). **^{13}C NMR** (101 MHz, chloroform-*d*) δ 178.72, 155.69, 137.06, 129.21, 129.01, 128.94, 128.62, 128.47, 128.33, 127.99, 127.53, 127.28, 120.61, 77.16, 67.72, 46.83, 45.93, 44.61, 41.43, 27.54, 24.53. **HRMS** (ESI-TOF) calcd for $C_{26}H_{27}O_3N_2$ $[M+H]^+$: 415.2016, found : 415.2020.

N-([1,1’-Biphenyl]-4-yl)piperidine-4-carboxamide 33

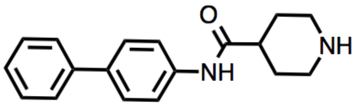

29 (960mg, 2.31 mmol) and palladium on carbon (24 mg, 0.23 mmol) were dissolved in MeOH (10 ml). The resultant reaction mixture was flushed with N_2 and removed in *vacuo* (repeated 3 times) before the addition of H_2 ballon. The resultant mixture was stirred for 16 hrs, before filtered through a Celite pad. The organic fraction was removed in *vacuo* to give **33** (490 mg, 1.75 mmol, 75 %) as a white solid.

m.p. 206 -208 °C. **IR** $_{νmax}$ (film) 3277, 2938, 1655 cm^{-1} . **^{1}H NMR** (400 MHz, DMSO-*d*$_6$) δ 9.92 (s, 1H, Amide NH), 8.02 – 6.97 (m, 9H, Ph), 2.98 (m, 3H), 2.48 – 2.33 (m, 1H), 1.74 - 1.52 (m, 6H). **^{13}C NMR** (101 MHz, DMSO-*d*$_6$) δ 173.85, 139.74, 138.97, 134.52, 128.88, 126.94, 126.81, 126.19, 119.39, 45.62, 43.76, 39.52, 29.47. **HRMS** (ESI-TOF) calcd for $C_{18}H_{21}N_2O$ $[M+H]^+$: 281.1648, found 281.1648.

N-([1,1'-Biphenyl]-4-ylmethyl)piperidine-4-carboxamide 34

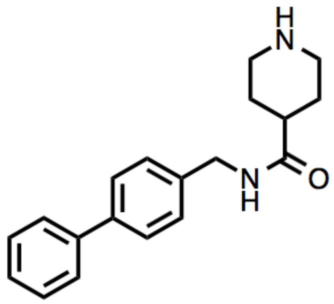

30 (376 mg, 0.87 mmol) and palladium on carbon (9.3 mg, 0.087 mmol) was dissolved in MeOH (10 ml). The resultant reaction mixture was flushed with N_2 and removed *in vacuo* (repeated 3 times) before the addition of H_2 ballon. The resultant mixture was stirred for 16 hrs, before filtered through a Celite pad. The organic fraction was removed in *vacuo* gave **34** (224 mg, 0.80 mmol, 87%) as a white solid.

m.p. 140 - 141 °C. **IR** ν_{max} (film) 3287, 2358, 1637 cm^{-1}. **^{1}H NMR** (400 MHz, chloroform-*d*) δ 7.61 – 7.30 (m, 9H, Ar), 4.47 (d, J = 5.5 Hz, 2H, CH_2), 3.14 (m, 2H, CH_2CH_2N), 2.62 (m, 2H, CH_2CH_2N), 2.27 (m, 1H, CH_2CH_2N), 1.93 – 1.81 (m, 2H, CH_2CH_2N), 1.74 – 1.56 (m, 2H, CH_2CH_2N). **^{13}C NMR** (101 MHz, Chloroform-*d*) δ 174.90, 140.77, 140.62, 137.51, 128.92, 128.62, 128.33, 128.29, 127.57, 127.49, 127.16, 46.09, 43.89, 43.27, 29.98. **HRMS** (ESI-TOF) calcd for $C_{19}H_{23}N_2O$ $[M+H]^+$: 295.1805, found 295.1803.

N-([1,1'-Biphenyl]-4-yl)piperidine-3-carboxamide 35

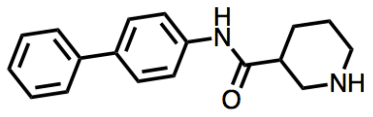

32 (611 mg, 1.376 mmol) and palladium on carbon (14.58 mg, 0.137 mmol) were dissolved in MeOH (10 ml). The resultant reaction mixture was flushed with N_2 and removed *in vacuo* (repeated 3 times) before the addition of H_2 ballon. The resultant mixture was stirred for 16 hrs, before filtered through a Celite pad. The organic fraction was removed in *vacuo* gave **35** (362 mg, 1.29 mmol, 94 %) as a white solid. **m.p.** >250°C. **IR** $_{\nu max}$ (film) 3332, 2361, 1652 cm^{-1}. **^{1}H NMR** (400 MHz, DMSO-*d*$_6$) δ 10.44 (s, 1H, NH), 7.82 – 7.57 (m, 6H, Ar), 7.54 – 7.31 (m, 3H, Ar), 3.38 – 2.69 (m, 6H, CH_2CH_2N), 2.11 – 1.50 (m, 3H, CH_2CH_2N). **^{13}C NMR** (101 MHz, DMSO-*d*$_6$) δ 170.86, 139.64, 138.42, 135.04, 128.94, 127.09, 126.92, 126.26, 119.62, 44.07, 43.90, 42.87, 39.52, 26.39, 21.17. **HRMS** (ESI-TOF) calcd for $C_{18}H_{21}ON_2$ $[M+H]^+$: 281.1648, found : 281.1644.

N-([1,1'-Biphenyl]-4-yl)-1-((3-methylpyridin-2-yl)methyl)piperidine-4-carboxamide 36

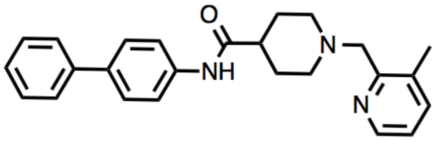

Following general procedure B: **33** (100 mg, 0.357 mmol), 3-methylpyridine-2-carboxaldehyde (80 µl, 0.714 mmol) and sodium triacetoxyborohydride (226 mg, 1.07 mmol) gave **36** (70 mg, 0.181 mmol, 51 %) as an brown oil.

IR ν_{max} (film) 2912, 1655 cm^{-1}. **^{1}H NMR** (400 MHz, DMSO-d_6) δ 9.97 (s, 1H, NH), 8.36 – 8.24 (m, 1H, Pyr), 7.77 – 7.07 (m, 11H, Ar), 3.58 (s, 2H, Pyr-<u>CH_2</u>), 2.39 (s, 3H, CH_3), 2.36 – 2.00 (m, 4H, CH_2CH_2N), 1.81 – 1.52 (m, 5H, CH_2CH_2N). **^{13}C NMR** (101 MHz, DMSO-d_6) δ 174.08, 146.06, 140.19, 139.33, 138.28, 135.05, 133.47, 129.33, 127.27, 126.65, 122.93, 119.88, 63.49, 53.40, 43.43, 29.03, 18.38. **HRMS** (ESI-TOF) calcd for $C_{25}H_{28}N_3O$ $[M+H]^+$: 386.2227, found 386.2226.

N-([1,1'-Biphenyl]-4-yl)-1-(2-hydroxybenzyl)piperidine-4-carboxamide 37

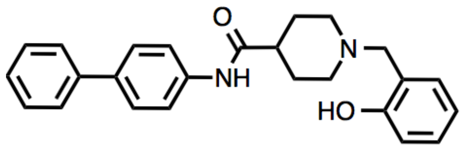

Following general procedure A: **33** (100 mg, 0.357 mmol), salicylicaldehyde (80 μl, 0.714 mmol) and sodium triacetoxyborohydride (226 mg, 1.07 mmol) gave **37** (63 mg, 0.163 mmol, 46 %) as a white solid.

m.p. 161-162 ℃. **IR** ν_{max} (film) 3233, 1668, 1505 cm^{-1}. **^{1}H NMR** (400 MHz, DMSO-d_6) δ 10.00 (s, 1H, NH), 7.70 - 7.10 (m, 11H, Ar), 6.81 – 6.69 (m, 2H, Phenol), 3.66 (s, 2H, CH_2), 2.97 (m, 2H, CH_2CH_2N), 2.42 (m, 1H, CH_2CH_2N), 2.10 (m, 2H, CH_2CH_2N), 1.89 – 1.62 (m, 5H, CH_2CH_2N). **^{13}C NMR** (101 MHz, DMSO-d_6) δ 173.77, 157.51, 140.17, 139.27, 135.12, 129.52, 129.35, 128.59, 127.30, 126.66, 122.72, 119.91, 119.19, 115.81, 59.79, 52.54, 43.03, 28.91. **HRMS** (ESI-TOF) calcd for $C_{25}H_{25}N_2O_2$, $[M-H]^-$: 385.1922, found 385.1926.

N-([1,1’-Biphenyl]-4-yl)-1-((3-hydroxypyridin-2-yl)methyl)piperidine-4-carboxamide 38

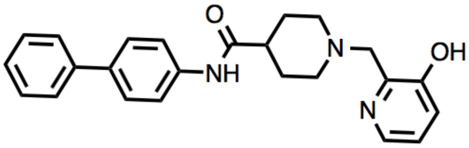

Following general procedure A: **33** (100 mg, 0.357 mmol), 3-hydroxypyridine-2-carboxaldehyde (81 mg, 0.714 mmol) and sodium triacetoxyborohydride (226 mg, 1.07 mmol) gave **38** (80 mg, 0.206 mmol, 57%) as a brown solid.

m.p. 197 - 198 °C. **IR** ν_{max} (film) 3421, 2917, 1686 cm^{-1}. **^{1}H NMR** (400 MHz, DMSO-d_6) δ 10.02 (s, 1H, NH), 7.95 –7.07 (m, 12H, Ar), 3.85 (s, 2H, CH_2), 2.98 (m, 2H, CH_2CH_2N), 2.43 (m, 1H, CH_2CH_2N), 2.21 (m, 2H, CH_2CH_2N), 1.86 (m, 2H, CH_2CH_2N), 1.69 (m, 2H, CH_2CH_2N). **^{13}C NMR** (101 MHz, DMSO-d_6) δ 173.67, 154.10, 143.57, 140.16, 139.82, 139.24, 135.15, 129.35, 127.44, 127.31, 126.66, 123.87, 122.65, 119.92, 62.64, 52.64, 42.73, 39.34, 28.83. **HRMS** (ESI-TOF) calcd for $C_{24}H_{24}N_3O_2$ $[M-H]^-$: 386.1874, found 386.1867.

N-([1,1’-Biphenyl]-4-ylmethyl)-1-((3-methylpyridin-2-yl)methyl)piperidine-4-carboxamide 39

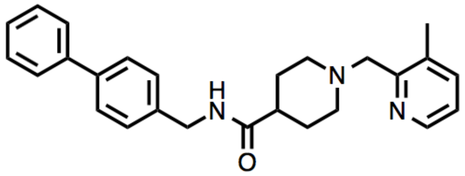

Following general procedure A: **34** (98 mg, 0.33 mmol), 3-methylpyridine-2-carboxaldehyde (37 µl, 0.33 mmol) and sodium triacetoxyborohydride (280 mg, 1.33 mmol) gave **39** (85 mg, 0.213 mmol, 64 %) as a yellow oil.

IR ν_{max} (film) 3195, 2254, 1664 cm^{-1}. **^{1}H NMR** (400 MHz, DMSO-d_6) δ 8.34 (t, J = 6.0 Hz, 1H, NH), 8.28 (m, 1H, Pyr), 7.68 – 7.18 (m, 11 H, Ar), 4.29 (d, J = 6.0 Hz, 2H, CH_2NH), 3.54 (s, 2H, CH_2Pyr), 2.78 (m, 2H, CH_2CH_2N), 2.37 (s, 3H, CH_3), 2.23 – 1.94 (m, 3H, CH_2CH_2N), 1.70 – 1.50 (m, 4H, CH_2CH_2N). **^{13}C NMR** (101 MHz, DMSO-d_6) δ 174.62, 171.67, 156.56, 145.62, 140.03, 139.06, 138.67, 137.85, 133.07, 128.97, 128.77, 127.99, 127.70, 127.35, 126.63, 126.60, 122.49, 118.74, 118.35, 103.13, 66.32, 66.10, 63.20, 53.06, 42.15, 38.89, 31.93, 28.79, 27.29, 23.22, 22.55, 17.96. **HRMS** (ESI-TOF) calcd for $C_{26}H_{30}N_3O$ [M+H]$^+$: 400.2383, found 400.2382.

N-([1,1'-Biphenyl]-4-ylmethyl)-1-(2-hydroxybenzyl)piperidine-4-carboxamide 40

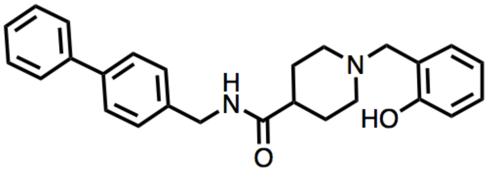

Following general procedure A: **34** (98 mg, 0.333 mmol), salicylicaldehyde (35 µl, 0.333 mmol) and sodium triacetoxyborohydride (278 mg, 1.32 mmol) gave **40** (58 mg, 0.145 mmol, 43 %) as a colourless oil.

IR ν_{max} (film) 3280, 1643 cm^{-1}. **^{1}H NMR** (400 MHz, DMSO-d_6) δ 8.38 (t, J = 6.0 Hz, 1H, NH), 7.66 – 6.74 (m, 12H), 4.31 (d, J = 6.0 Hz, 2H, CH_2NH), 3.63 (s, 2H, $\underline{CH_2}$-Phenol), 2.92 (m, 2H, CH_2CH_2N), 2.26 (m, 1H, CH_2CH_2N), 2.06 (m, 2H, CH_2CH_2N), 1.88 – 1.52 (m, 4H, CH_2CH_2N). **^{13}C NMR** (101 MHz, DMSO-d_6) δ 174.15, 157.12, 139.96, 138.96, 138.63, 129.00, 128.91, 128.42, 128.13, 127.66, 127.50, 127.30, 126.59, 126.55, 122.15, 118.70, 115.34, 66.13, 59.43, 52.11, 43.12, 41.61, 28.55. **HRMS** (ESI-TOF) calcd for $C_{26}H_{28}N_2O_2$ [M+H]$^+$: 401.2224, found 401.2223.

N-([1,1'-Biphenyl]-4-ylmethyl)-1-((3-hydroxypyridin-2-yl)methyl)piperidine-4-carboxamide 41

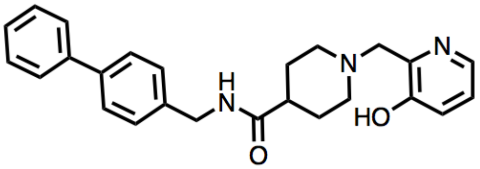

Following general procedure A: **34** (98 mg, 0.33 mmol), 3-hydroxypyridine-2-carboxaldehyde (81 mg, 0.66 mmol) and sodium triacetoxyborohydride (280 mg, 1.33 mmol) gave **41** (60 mg, 0.15 mmol, 45 %) as a white solid.

m.p. 211 - 212 ℃. **IR** $_{\nu max}$ (film) 3261, 1668 cm^{-1}. ^{1}**H NMR** (400 MHz, DMSO-d_6) δ 8.40 (t, J = 6.0 Hz, 1H, NH), 7.94 (m, 1H, Pyr), 7.72 – 7.04 (m, 11H, Ar), 4.31 (d, J = 6.0 Hz, 2H, <u>CH_2</u>NH), 3.83 (s, 2H, CH_2Pyr), 2.34 – 2.06 (m, 4H, CH_2CH_2N), 1.86 – 1.56 (m, 5H, $CHCH_2CH_2N$). ^{13}**C NMR** (101 MHz, DMSO-d_6) δ 174.53, 154.16, 143.52, 140.42, 139.79, 139.40, 139.10, 129.38, 128.13, 127.77, 127.06, 127.02, 123.84, 122.63, 62.75, 52.69, 42.08, 41.78, 28.94. **HRMS** (ESI-TOF) calcd for $C_{25}H_{27}N_3O_2$ [M-H]$^-$: 400.2031, found 400.2027.

N-([1,1'-Biphenyl]-4-yl)-1-((3-methylpyridin-2-yl)methyl)piperidine-3-carboxamide 42

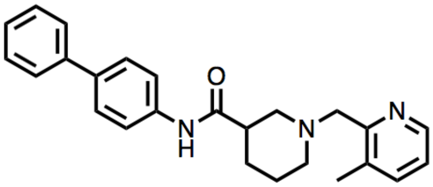

Following general procedure A: **35** (38 mg, 0.137 mmol), 3-methyl-pyridine-2-carboxaldehyde (33 mg, 0.275 mmol) and sodium triacetoxyborohydride (87 mg, 0.41 mmol) gave **42** (7 mg, 0.0181 mmol, 14 %) as an clear oil.

IR $_{\nu max}$ (film) 3562, 1664 cm^{-1} . **^{1}H NMR** (400 MHz, DMSO-d_6) δ 10.18 (s, 1H, NH), 8.33 – 8.28 (s 1H, Pyr), 7.89 – 6.95 (m, 11H, Ar), 3.61 (s, 2H, CH_2), 2.85 – 2.65 (m, 3H, CH_2CH_2N), 2.38 (s, 3H, CH_3), 2.36 – 2.11 (m, 1H, CH_2CH_2N), 1.88 – 1.36 (m, 5H, CH_2CH_2N). **^{13}C NMR** (101 MHz, DMSO-d_6) δ 172.57, 145.76, 139.72, 138.69, 137.87, 134.69, 132.80, 128.89, 126.98, 126.83, 126.21, 122.46, 119.53, 62.68, 55.70, 53.60, 43.41, 39.52, 27.21, 24.15, 17.97. **HRMS** (ESI-TOF) calcd for $C_{25}H_{28}ON_3$ $[M+H]^+$: 386.2227, found : 386.2222.

N-([1,1'-Biphenyl]-4-yl)-1-(2-hydroxybenzyl)piperidine-3-carboxamide 43

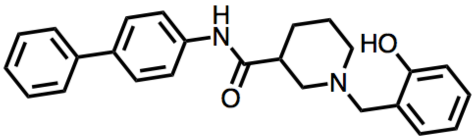

Following general procedure A: **35** (38 mg, 0.137 mmol), salicaldehyde (33 mg, 0.275 mmol) and sodium triacetoxyborohydride (87 mg, 0.41 mmol) gave **43** (10 mg, 0.026 mmol, 19 %) as a colourless oil.

IR $_{\nu max}$ (film) 3562, 1664 cm^{-1}. **^{1}H NMR** (400 MHz, DMSO-d_6) δ 10.09 (s, 1H, NH), 7.73 – 7.56 (m, 6H, Ar), 7.49 – 7.39 (m, 2H, Ar), 7.36 – 7.28 (m, 1H, Ar), 7.10 (m, 2H, Ar), 6.81 – 6.70 (m, 2H, Ar), 3.65 (s, 2H, CH_2), 2.97 (m, 1H, CH_2CH_2N), 2.87 – 2.76 (m, 1H, CH_2CH_2N), 2.69 – 2.60 (m, 1H, CH_2CH_2N), 2.37 – 2.24 (m, 1H, CH_2CH_2N), 2.13 – 2.05 (m, 1H, CH_2CH_2N), 1.89 – 1.84 (m, 1H, CH_2CH_2N), 1.78 – 1.67 (m, 1H, CH_2CH_2N), 1.57 – 1.46 (m, 2H, CH_2CH_2N). **^{13}C NMR** (101 MHz, DMSO-d_6) δ 172.13, 156.93, 139.70, 138.61, 134.78, 129.34, 128.87, 128.21, 127.28, 126.97, 126.82, 126.20, 122.26, 119.54, 118.75, 114.50, 59.23, 55.17, 52.49, 43.43, 39.52, 27.10, 24.20, 21.07. **HRMS** (ESI-TOF) calcd for $C_{25}H_{27}O_2N_2$ $[M+H]^+$: 387.2067, found : 387.2068

N-([1,1'-Biphenyl]-4-yl)-1-((3-hydroxypyridin-2-yl)methyl)piperidine-3-carboxamide 44

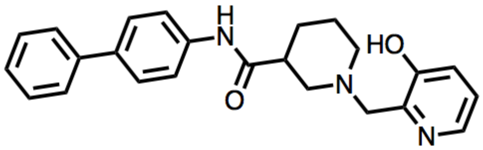

Following general procedure A: **35** (38 mg, 0.137 mmol), 3-hydroxypyridine-2-carboxaldehyde (33 mg, 0.275 mmol) and sodium triacetoxyborohydride (87 mg, 0.41 mmol) gave **44** (7.5 mg, 0.019 mmol, 14%) as a colourless oil.

IR ν_{max} (film) 3562, 1664 cm^{-1}. **^{1}H NMR** (400 MHz, DMSO-d_6) δ 10.20 (s, 1H, NH), 7.96 (m, 1H, Ar), 7.73 – 7.58 (m, 6H, Ar), 7.47 – 7.39 (m, 2H, Ar), 7.35 – 7.28 (m, 1H, Ar), 7.18 – 7.08 (m, 2H, Ar), 3.96 – 3.71 (s, 2H, CH_2), 3.00 – 2.91 (m, 1H, CH_2CH_2N), 2.77 (m, 1H, CH_2CH_2N), 2.65 (m, 1H, CH_2CH_2N), 2.45 (m, 1H, CH_2CH_2N), 2.29 (m, 1H, CH_2CH_2N), 1.85 (m, 1H, CH_2CH_2N), 1.80 – 1.70 (m, 1H, CH_2CH_2N), 1.55 (m, 2H, CH_2CH_2N). **^{13}C NMR** (101 MHz, DMSO-d_6) δ 172.09, 153.34, 143.38, 139.72, 139.42, 138.61, 134.82, 128.89, 126.99, 126.85, 126.22, 123.43, 122.19, 119.61, 61.53, 55.07, 52.76, 43.14, 39.52, 26.91, 23.95, 21.09. **HRMS** (ESI-TOF) calcd for $C_{24}H_{26}O_2N_3$ $[M+H]^+$: 388.2020, found : 388.2023.

Representative NMR spectra

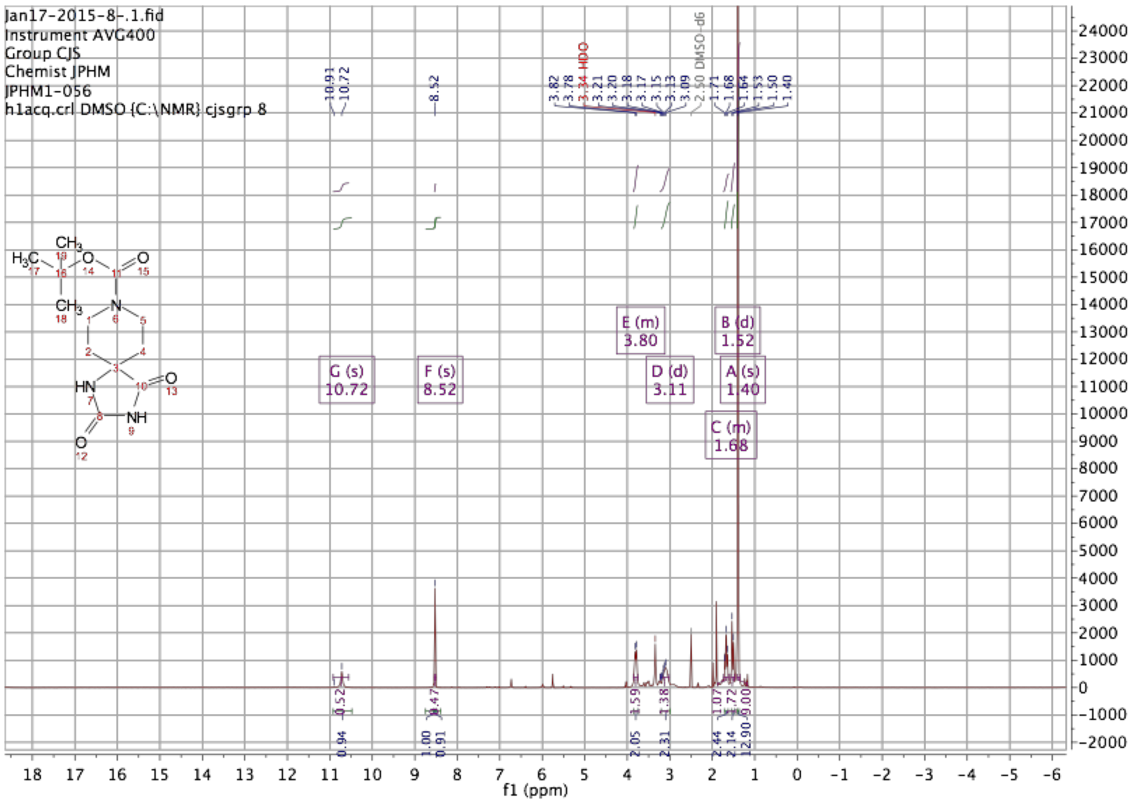

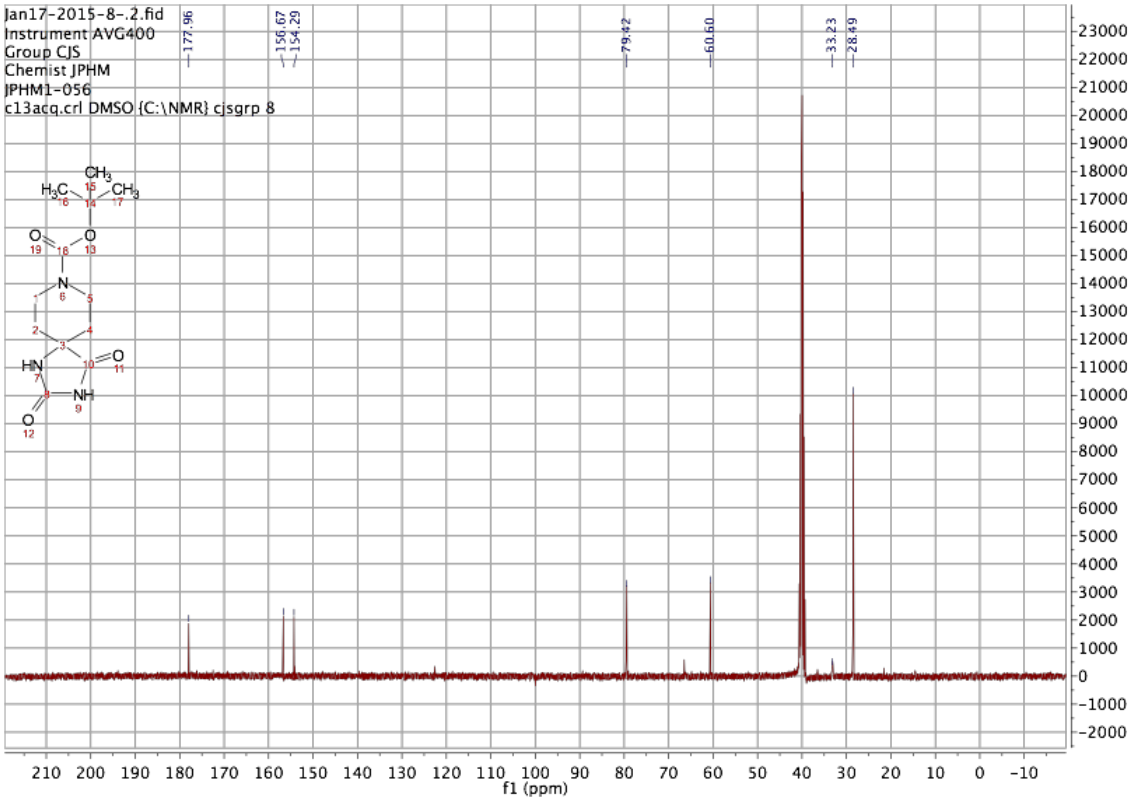

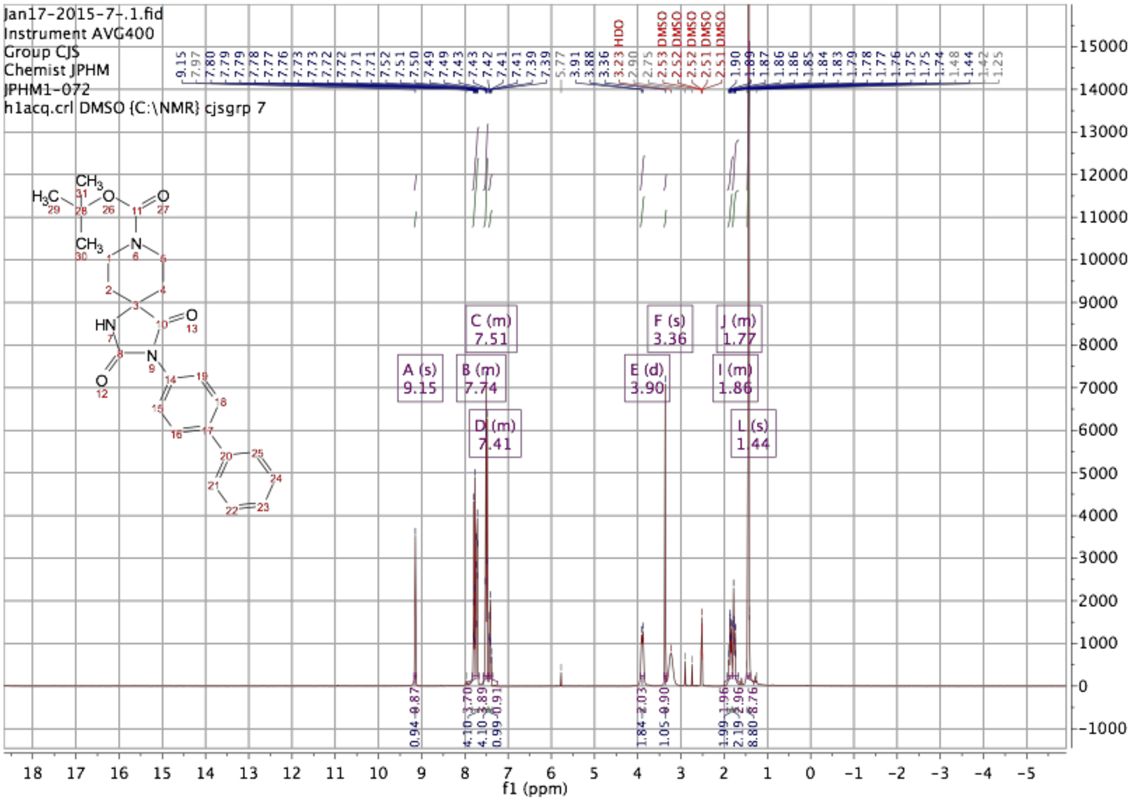
Jan17-2015-7-.1.fid
Instrument AVG400
Group CJS
Chemist JPHM
JPHM1-072
h1acq.crl DMSO {C:\NMR} cjsgrp 7
A (s) 9.15
B (m) 7.74
C (m) 7.51
D (m) 7.41
E (d) 3.90
F (s) 3.36
I (m) 1.86
J (m) 1.77
L (s) 1.44
3.23 HDO
2.53 DMSO
2.52 DMSO
2.52 DMSO
2.51 DMSO
2.51 DMSO
f1 (ppm)

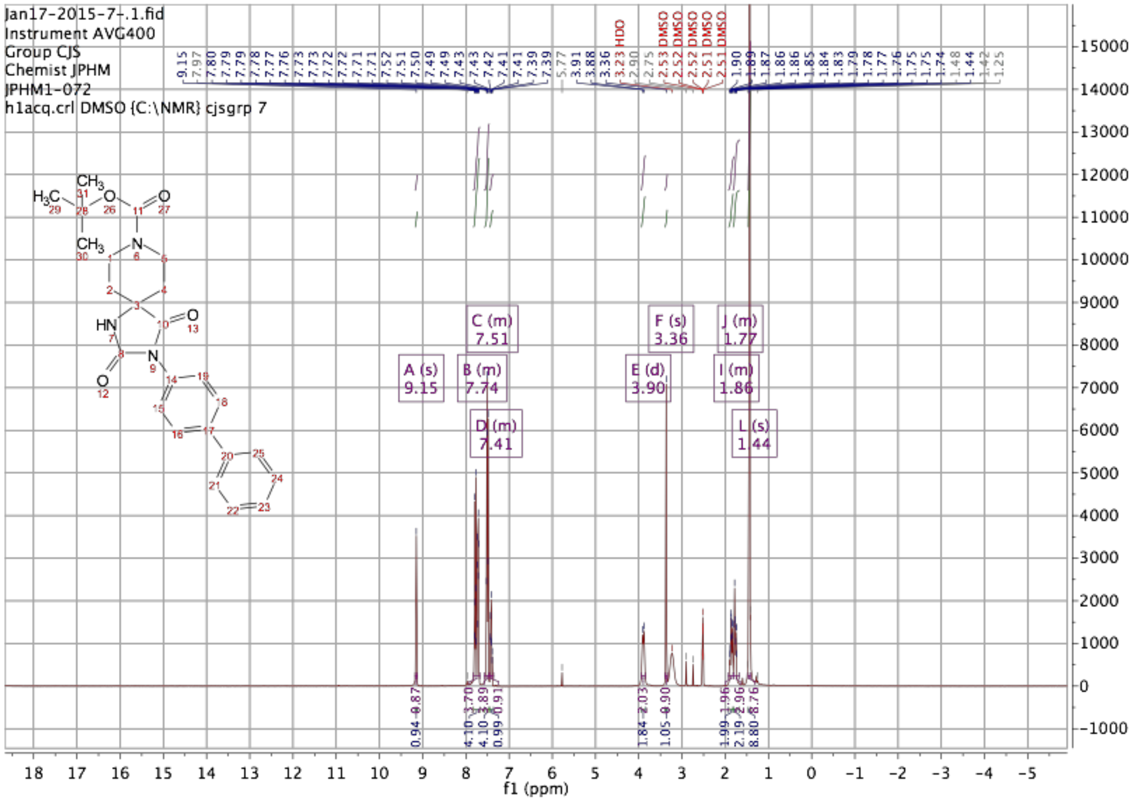
Jan17-2015-7-.1.fid
Instrument AVG400
Group CJS
Chemist JPHM
JPHM1-072
h1acq.crl DMSO {C:\NMR} cjsgrp 7
A (s) 9.15
B (m) 7.74
C (m) 7.51
D (m) 7.41
E (d) 3.90
F (s) 3.36
I (m) 1.86
J (m) 1.77
L (s) 1.44
3.23 HDO
2.53 DMSO
2.52 DMSO
2.52 DMSO
2.51 DMSO
2.51 DMSO
f1 (ppm)

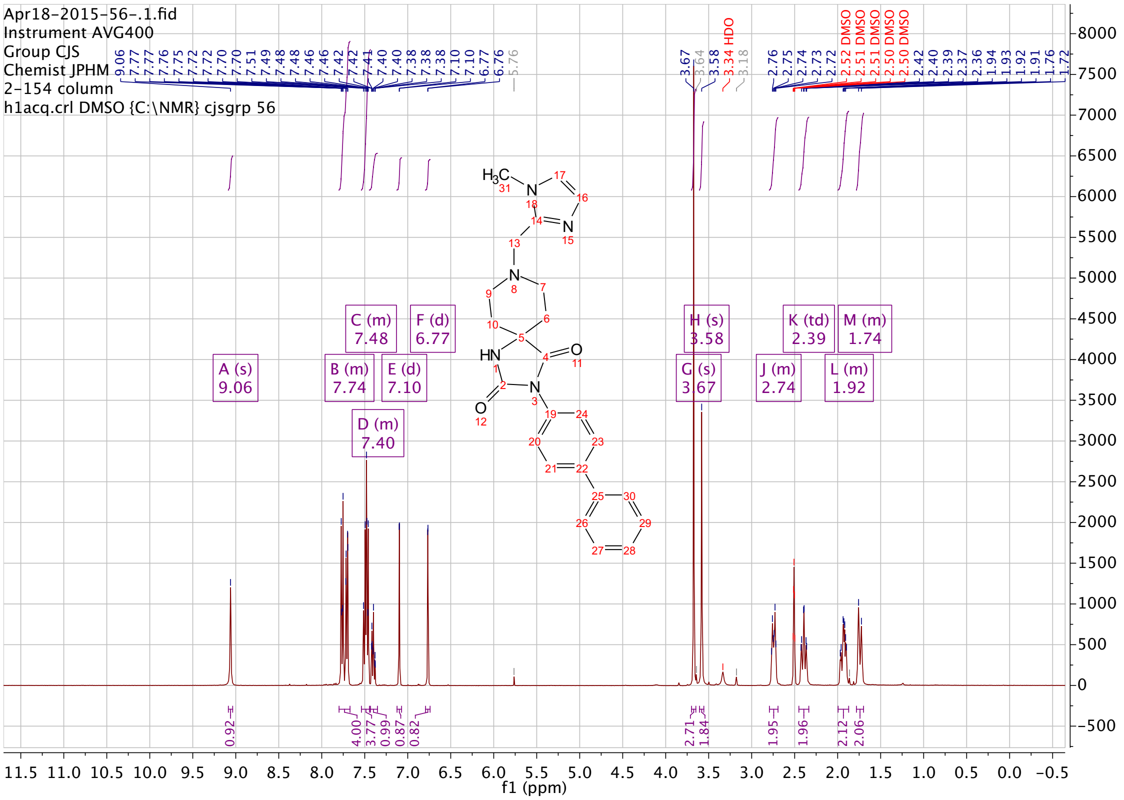
Apr18-2015-56-.1.fid
Instrument AVG400
Group CJS
Chemist JPHM
2-154 column
h1acq.crl DMSO {C:\NMR} cjsgrp 56
9.06 7.77 7.77 7.76 7.75 7.72 7.72 7.72 7.70 7.51 7.49 7.48 7.48 7.46 7.46 7.42 7.42 7.41 7.40 7.40 7.38 7.38 7.38 7.10 7.10 6.77 6.76 5.76
3.67 3.64 3.58 3.34 HDO 3.18
2.76 2.75 2.74 2.73 2.72 2.52 DMSO 2.51 DMSO 2.51 DMSO 2.50 DMSO 2.50 DMSO 2.42 2.40 2.39 2.37 2.36 1.94 1.93 1.92 1.91 1.76 1.72
A (s) 9.06
B (m) 7.74
C (m) 7.48
D (m) 7.40
E (d) 7.10
F (d) 6.77
G (s) 3.67
H (s) 3.58
J (m) 2.74
K (td) 2.39
L (m) 1.92
M (m) 1.74
0.92 4.00 3.77 0.99 0.87 0.82 2.71 1.84 1.95 1.96 2.12 2.06
f1 (ppm)

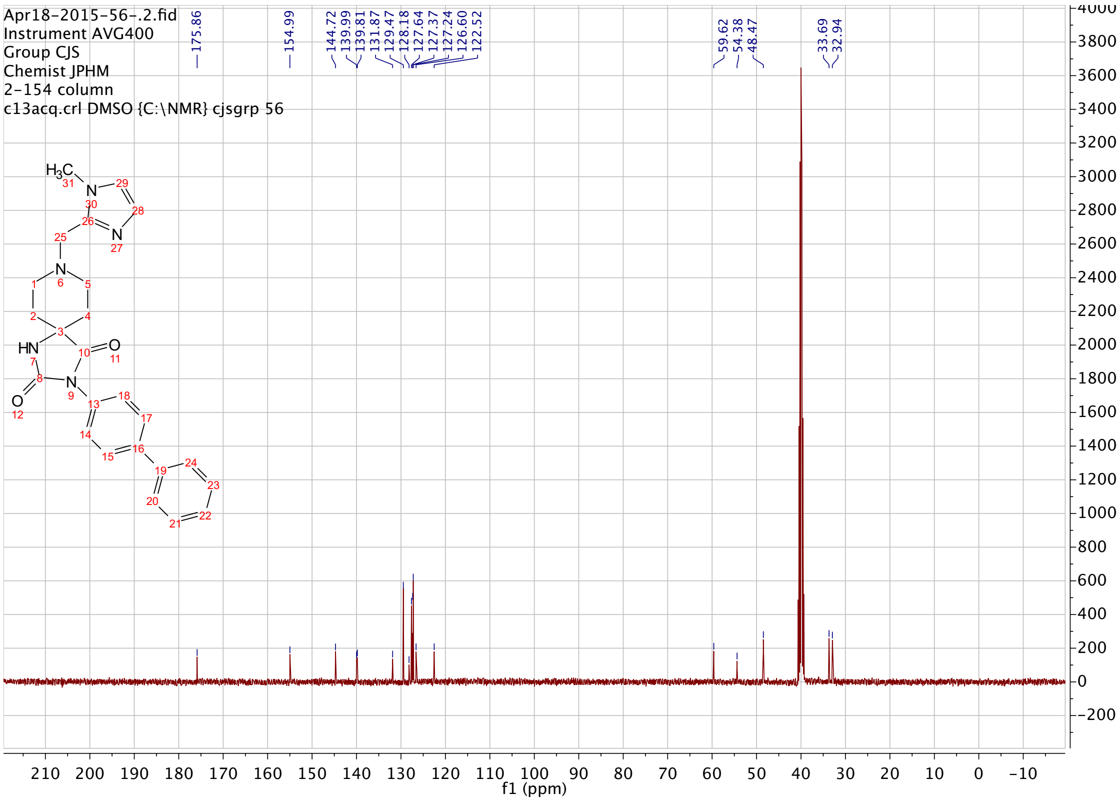
Apr18-2015-56-.2.fid
Instrument AVG400
Group CJS
Chemist JPHM
2-154 column
c13acq.crl DMSO {C:\NMR} cjsgrp 56
175.86
154.99
144.72 139.99 139.81 131.87 129.47 128.18 127.64 127.37 127.24 126.60 122.52
59.62 54.38 48.47
33.69 32.94
f1 (ppm)

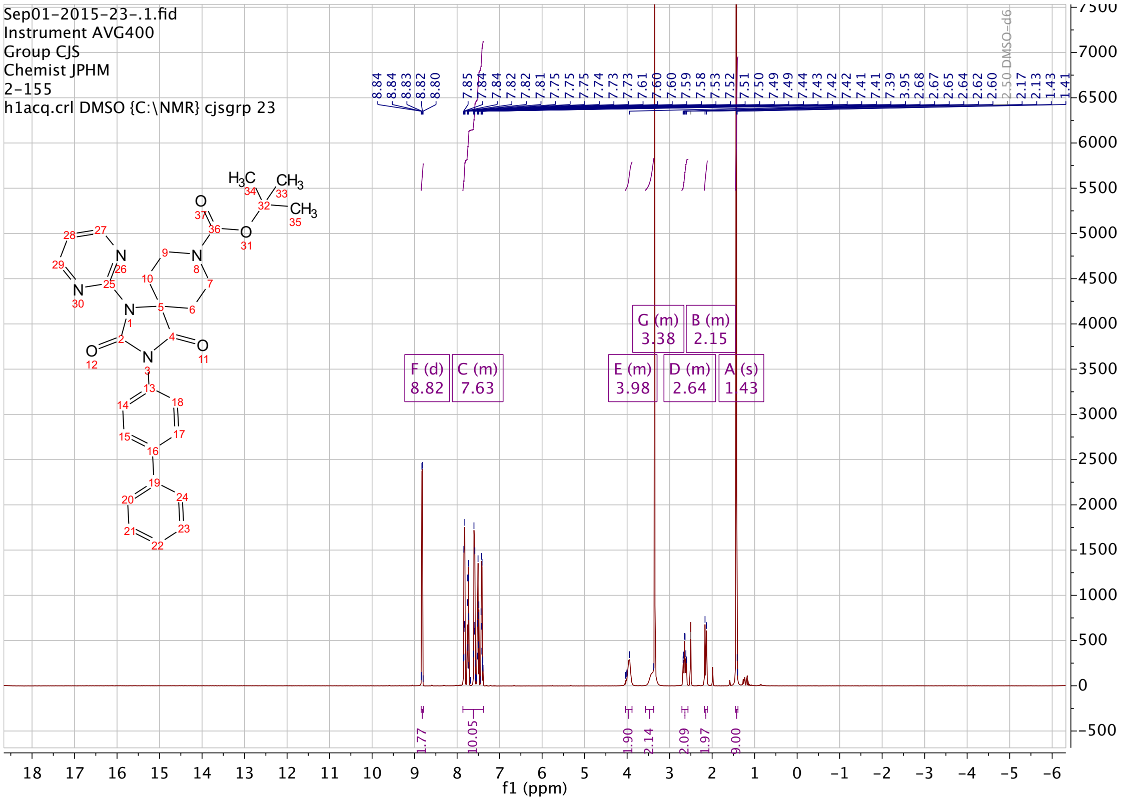
Sep01-2015-23-.1.fid
Instrument AVG400
Group CJS
Chemist JPHM
2-155
h1acq.crl DMSO {C:\NMR} cjsgrp 23
F (d) 8.82
C (m) 7.63
G (m) 3.38
B (m) 2.15
E (m) 3.98
D (m) 2.64
A (s) 1.43
f1 (ppm)

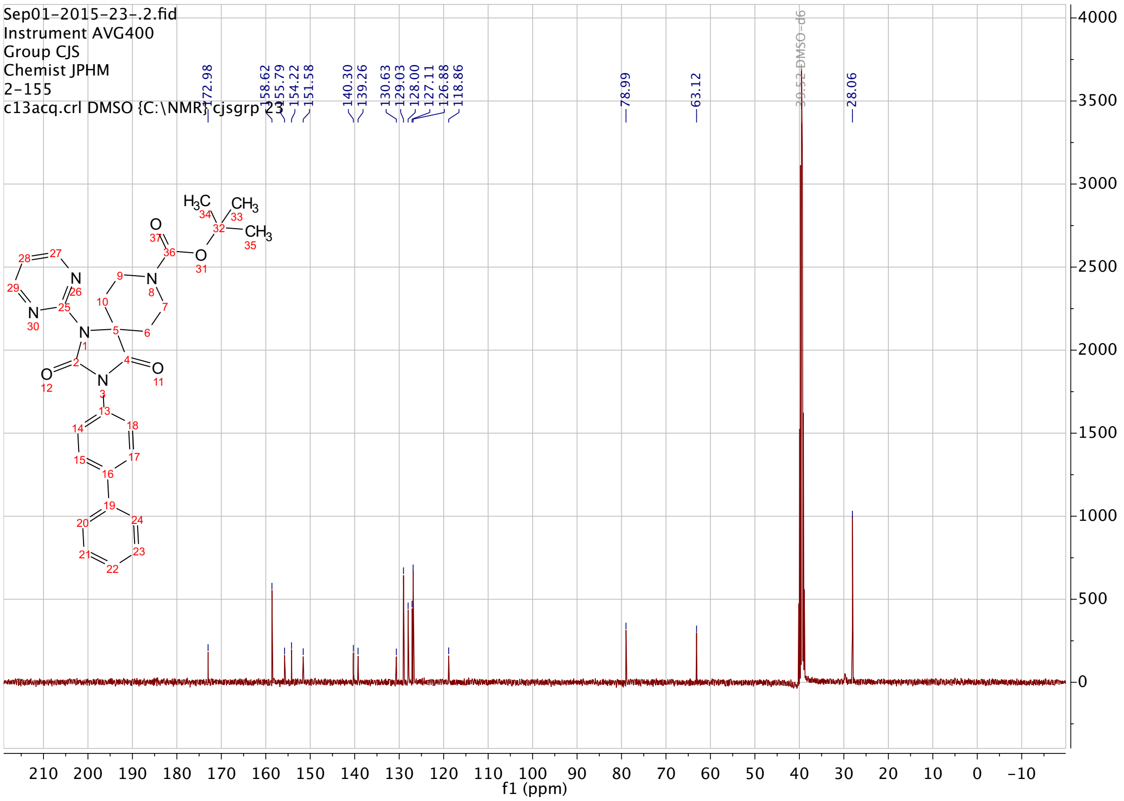
Sep01-2015-23-.2.fid
Instrument AVG400
Group CJS
Chemist JPHM
2-155
c13acq.crl DMSO {C:\NMR} cjsgrp 23
172.98
158.62
155.79
154.22
151.58
140.30
139.26
130.63
129.03
128.00
127.11
126.88
118.86
78.99
63.12
39.52 DMSO-d6
28.06
f1 (ppm)

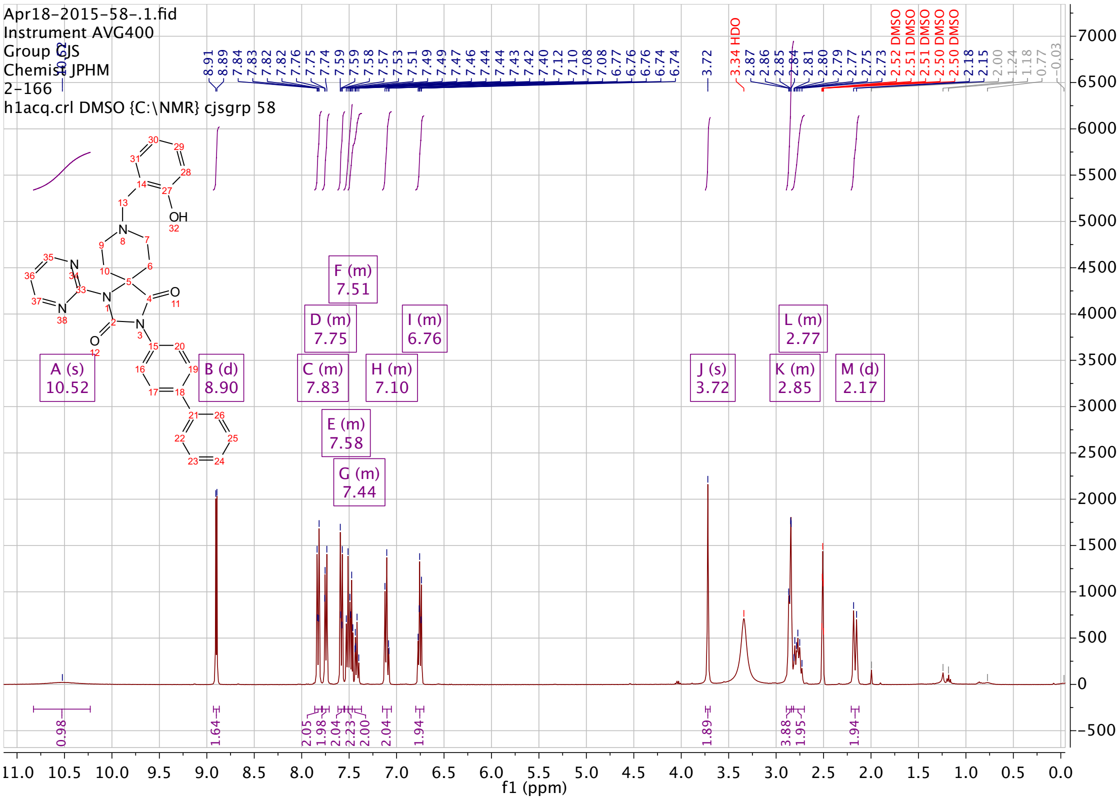
Apr18-2015-58-.1.fid
Instrument AVG400
Group CJS
Chemist JPHM
2-166
h1acq.crl DMSO {C:\NMR} cjsgrp 58
A (s) 10.52
B (d) 8.90
C (m) 7.83
D (m) 7.75
E (m) 7.58
F (m) 7.51
G (m) 7.44
H (m) 7.10
I (m) 6.76
J (s) 3.72
K (m) 2.85
L (m) 2.77
M (d) 2.17
f1 (ppm)

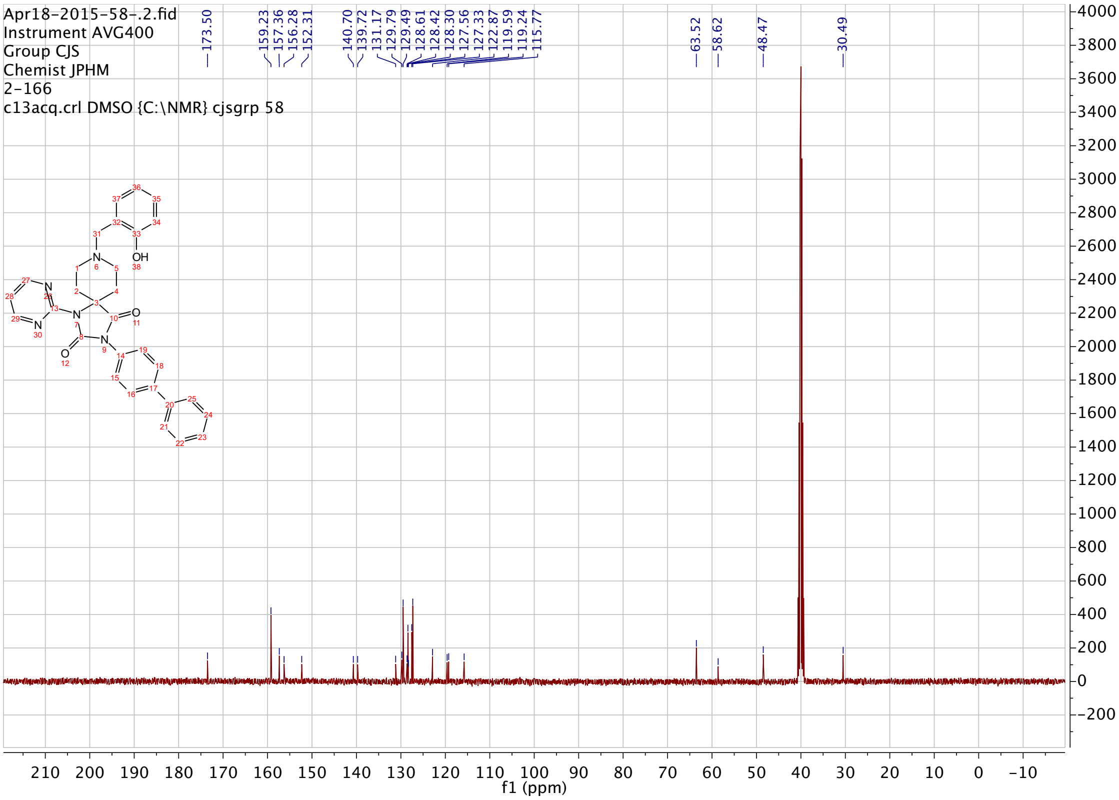
Apr18-2015-58-.2.fid
Instrument AVG400
Group CJS
Chemist JPHM
2-166
c13acq.crl DMSO {C:\NMR} cjsgrp 58
173.50
159.23
157.36
156.28
152.31
140.70
139.72
131.17
129.79
129.49
128.61
128.42
128.30
127.56
127.33
122.87
119.59
119.24
115.77
63.52
58.62
48.47
30.49
f1 (ppm)

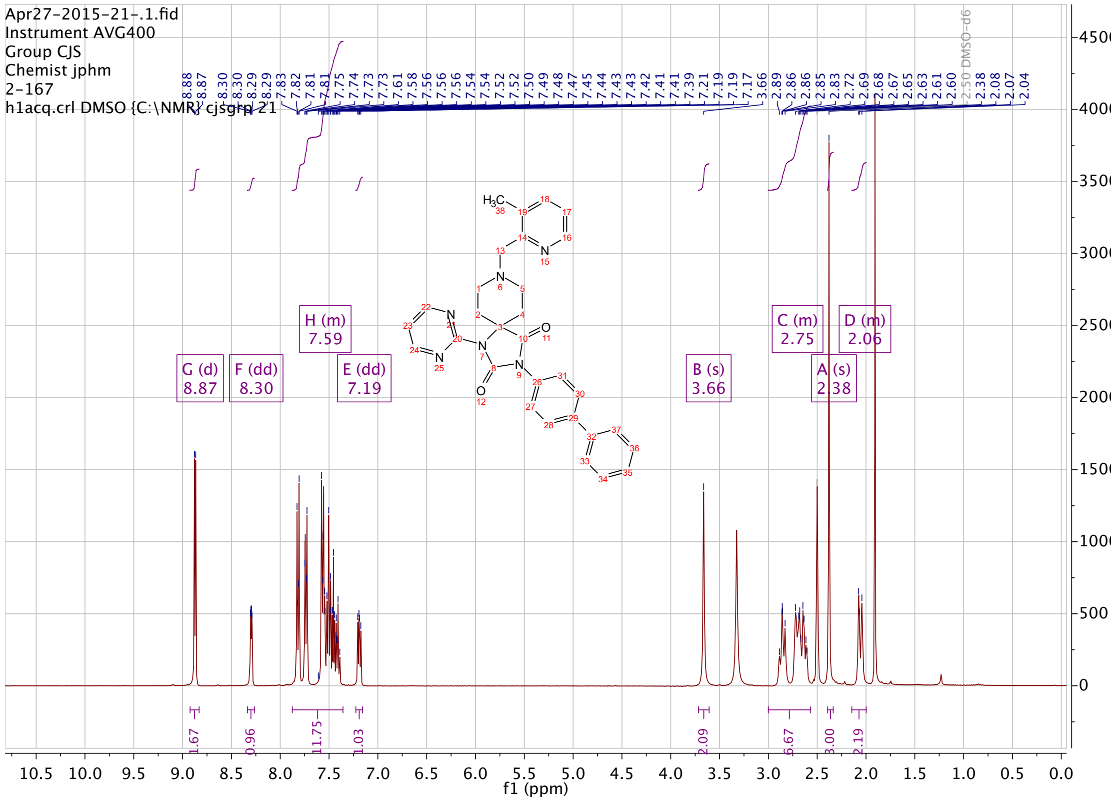
Apr27-2015-21-.1.fid
Instrument AVG400
Group CJS
Chemist jphm
2-167
h1acq.crl DMSO {C:\NMR} cjsgrp 21
G (d) 8.87
F (dd) 8.30
H (m) 7.59
E (dd) 7.19
B (s) 3.66
C (m) 2.75
D (m) 2.06
A (s) 2.38
2.50 DMSO-d6
f1 (ppm)

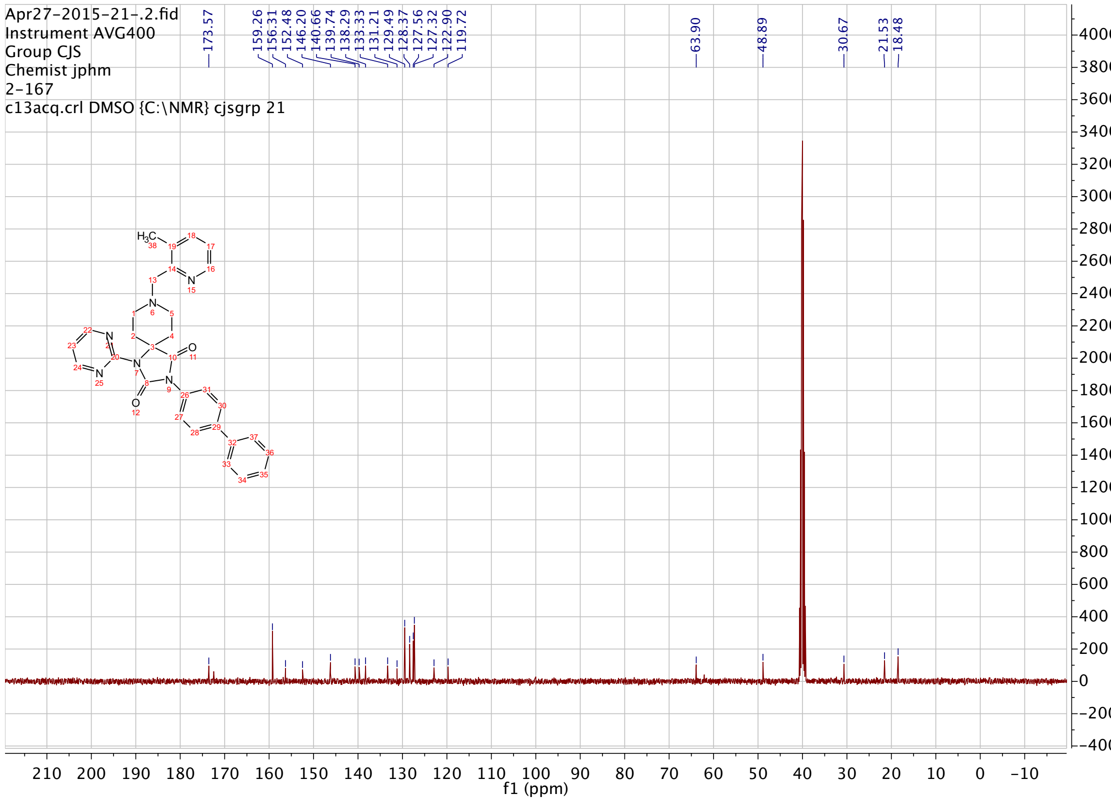
Apr27-2015-21-.2.fid
Instrument AVG400
Group CJS
Chemist jphm
2-167
c13acq.crl DMSO {C:\NMR} cjsgrp 21
173.57
159.26
156.31
152.48
146.20
140.66
139.74
138.29
133.33
131.21
129.49
128.37
127.56
127.32
122.90
119.72
63.90
48.89
30.67
21.53
18.48
f1 (ppm)

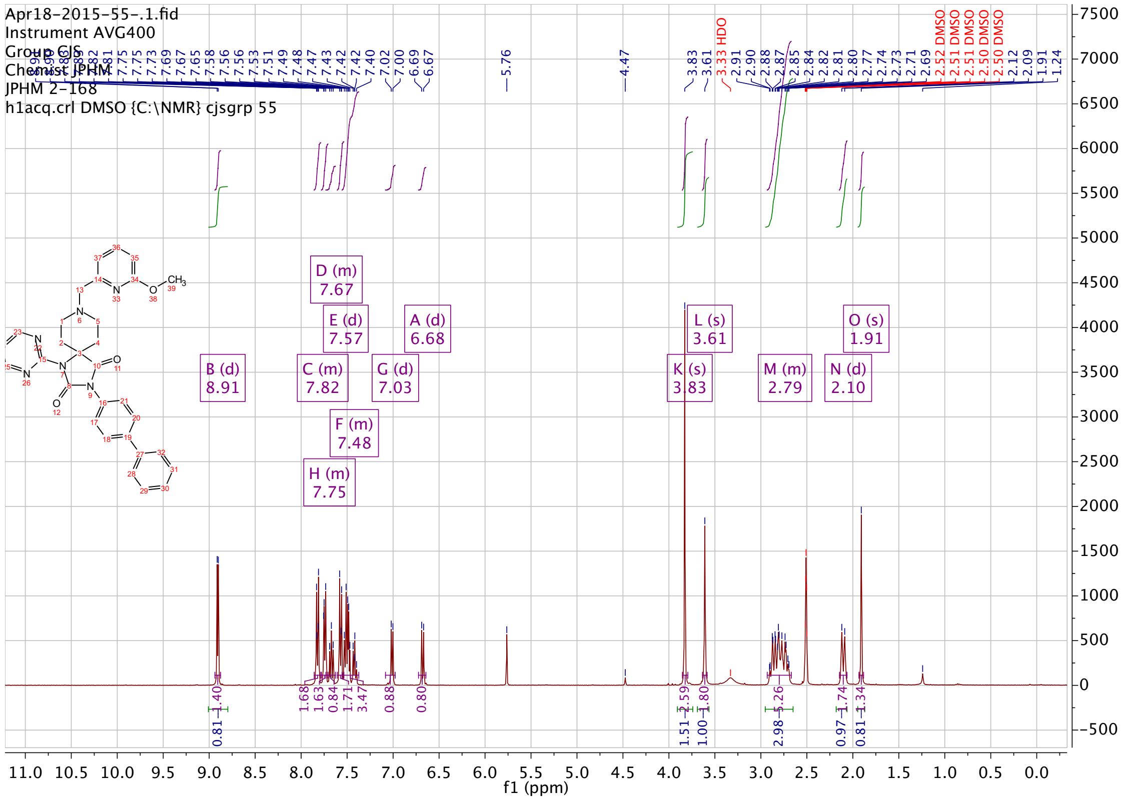
Apr18-2015-55-.1.fid
Instrument AVG400
Group CJS
Chemist JPHM
JPHM 2-168
h1acq.crl DMSO {C:\NMR} cjsgrp 55
f1 (ppm)

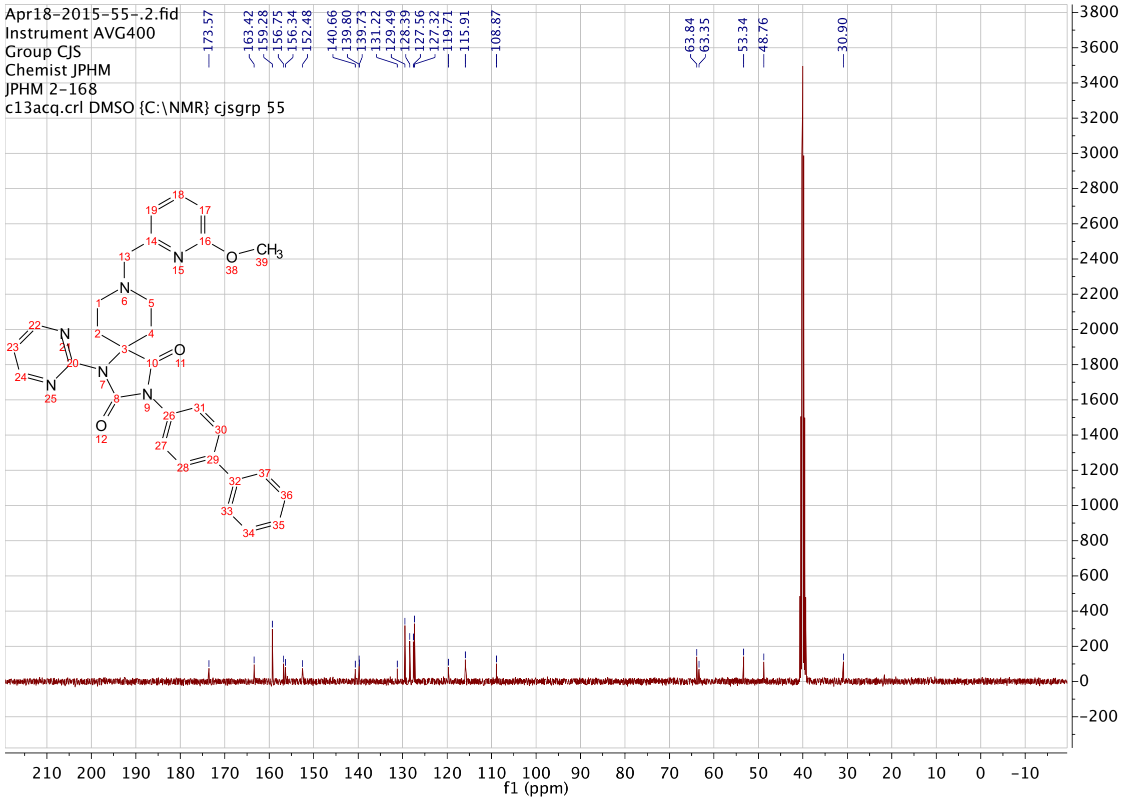
Apr18-2015-55-.2.fid
Instrument AVG400
Group CJS
Chemist JPHM
JPHM 2-168
c13acq.crl DMSO {C:\NMR} cjsgrp 55
f1 (ppm)

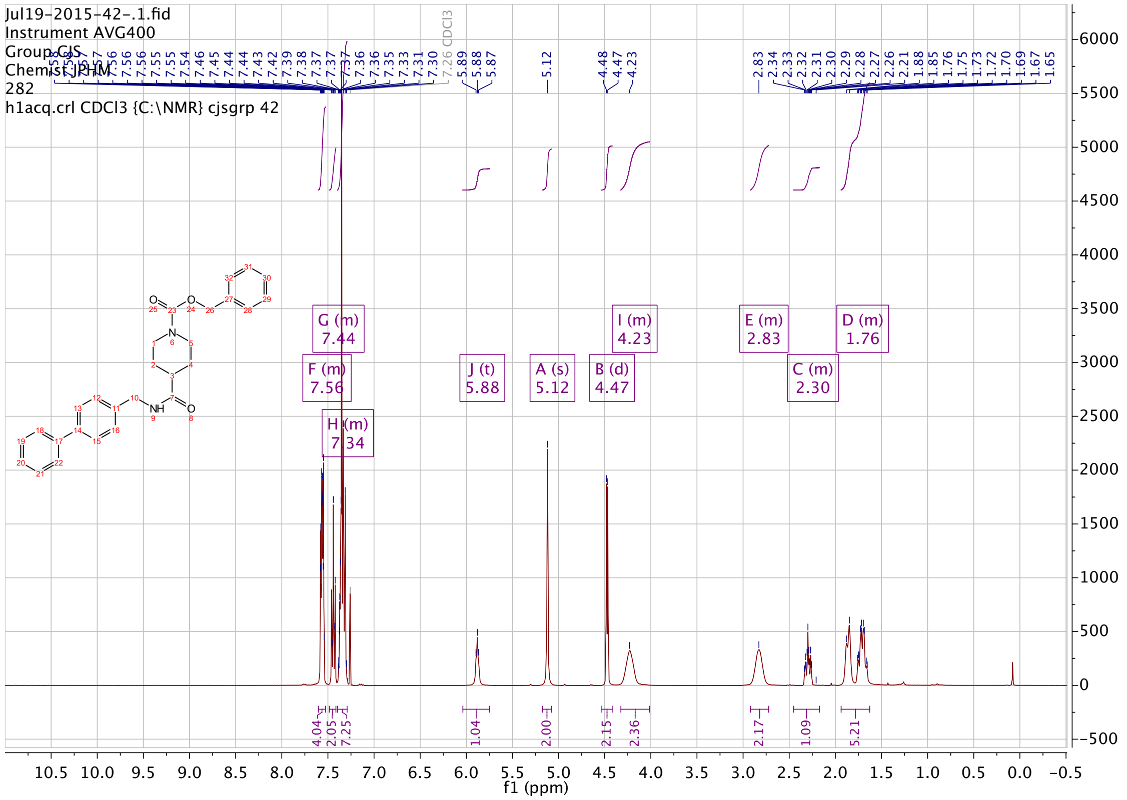

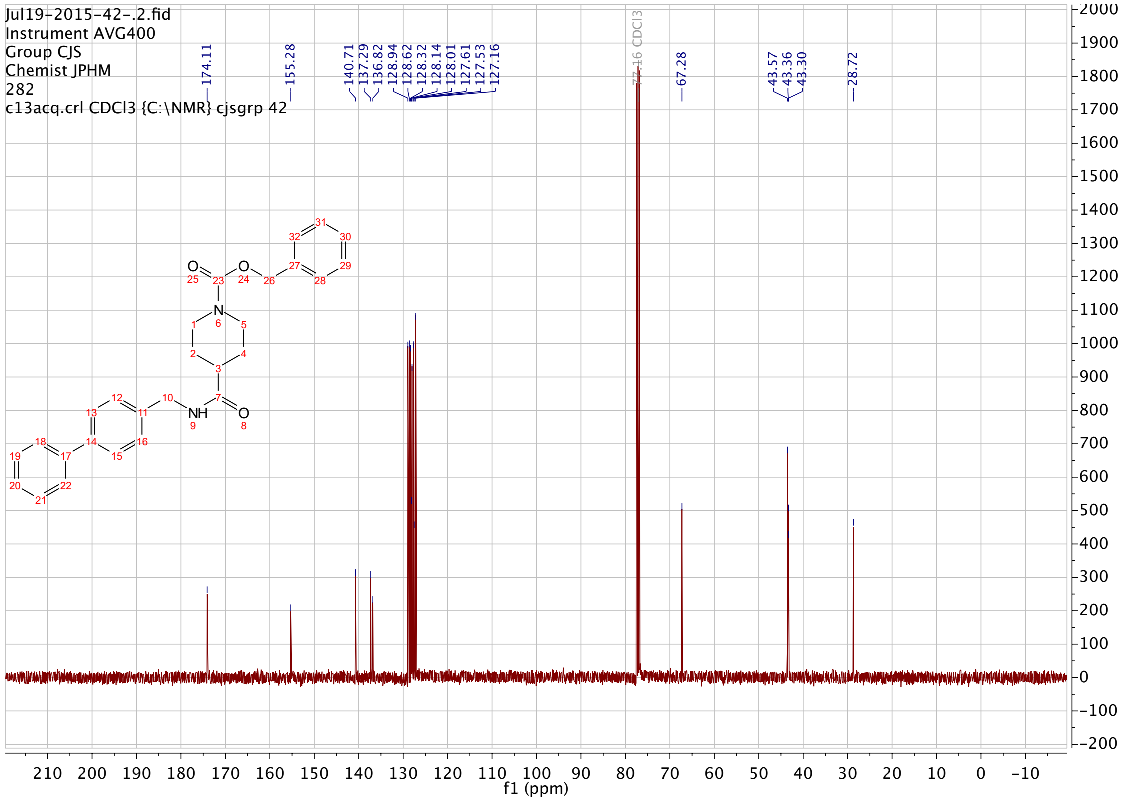

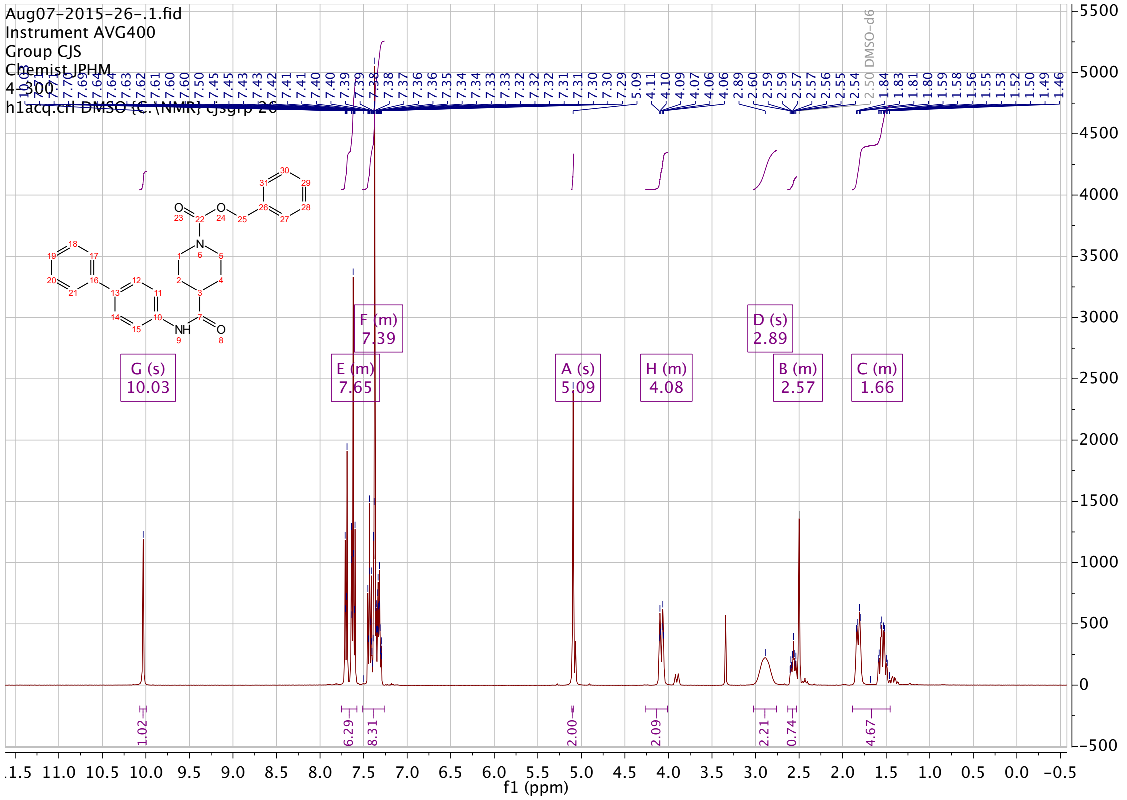

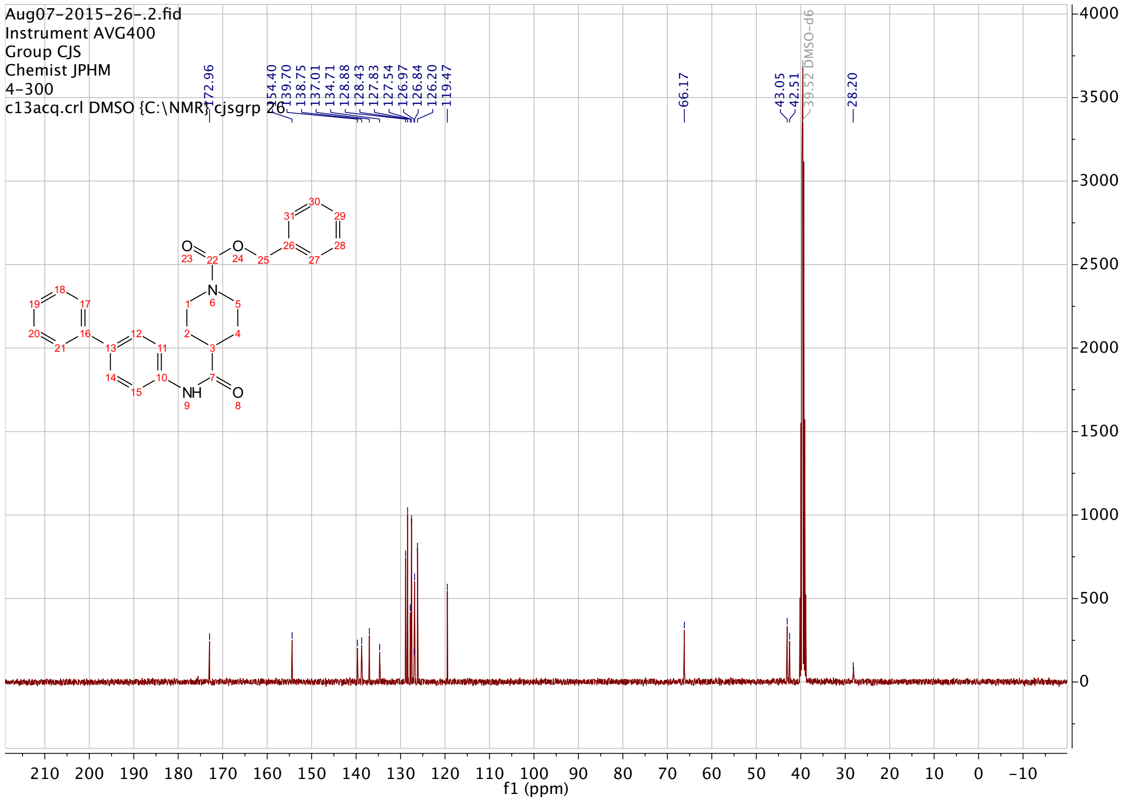

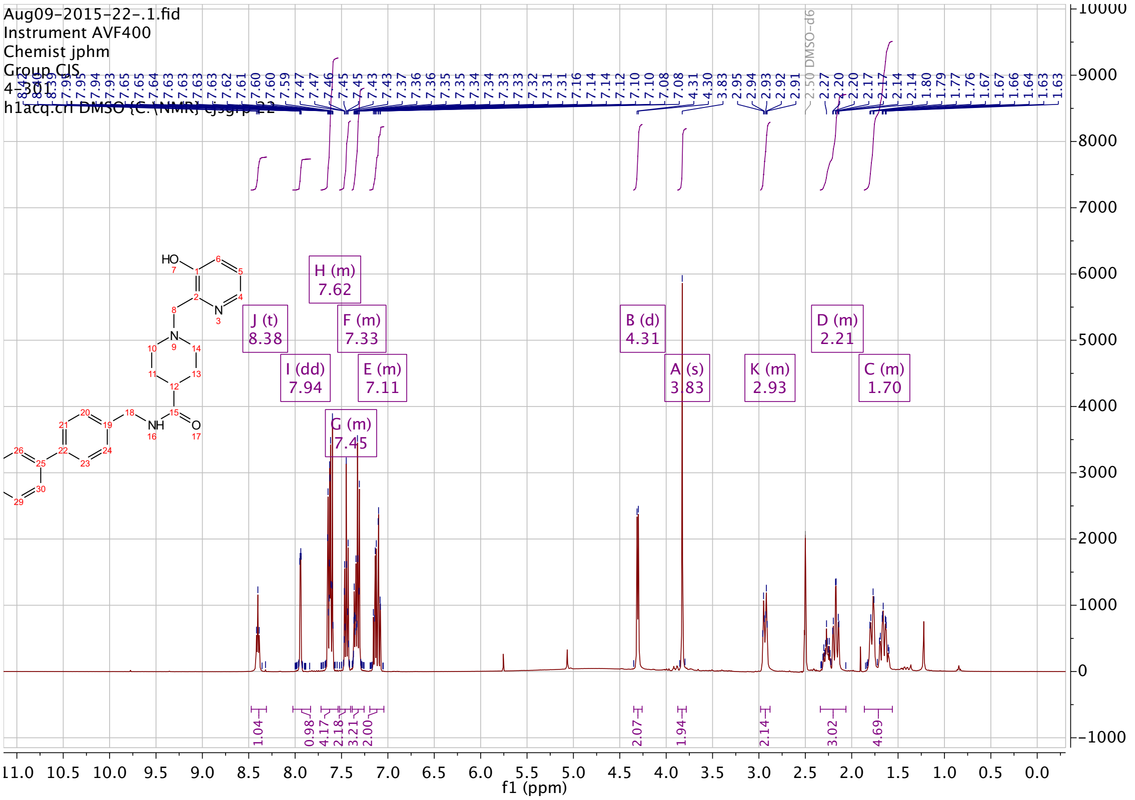
Aug09-2015-22-.1.fid
Instrument AVF400
Chemist jphm
Group CJS
4-301
h1acq.crl DMSO {C:\NMR} cjsgrp 22
J (t) 8.38
I (dd) 7.94
H (m) 7.62
G (m) 7.45
F (m) 7.33
E (m) 7.11
B (d) 4.31
A (s) 3.83
K (m) 2.93
D (m) 2.21
C (m) 1.70
2.50 DMSO-d6
f1 (ppm)

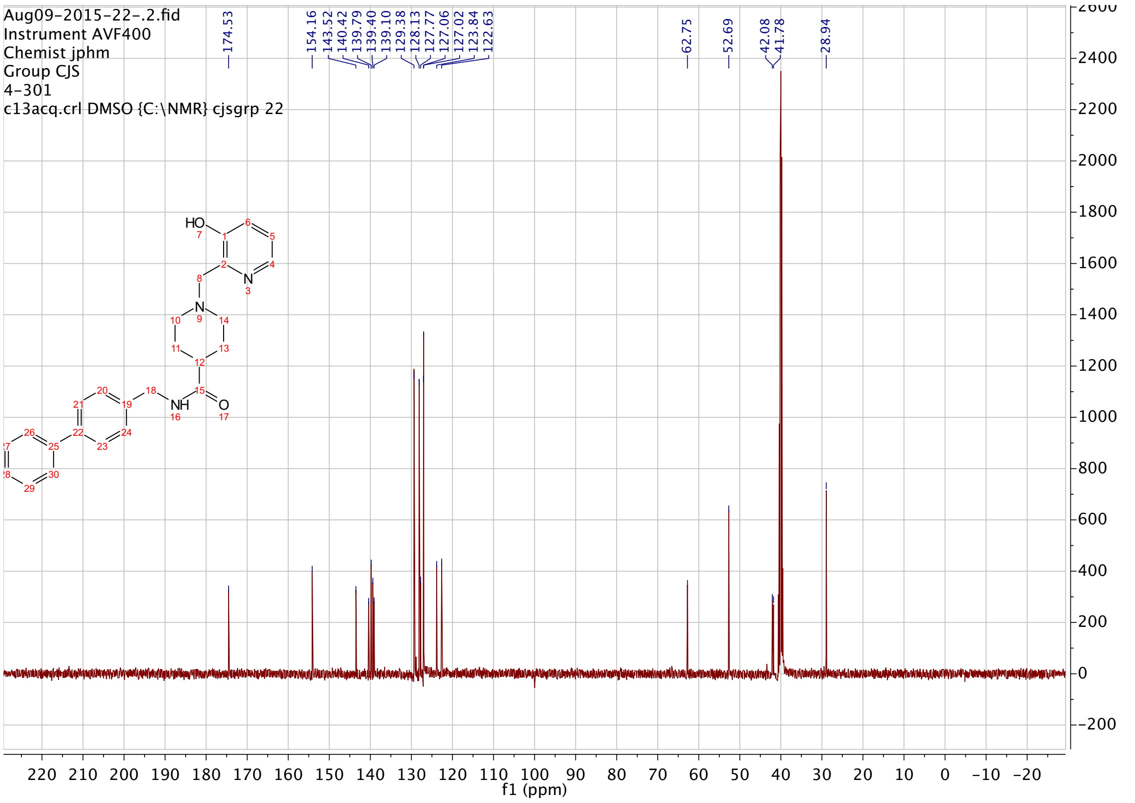
Aug09-2015-22-.2.fid
Instrument AVF400
Chemist jphm
Group CJS
4-301
c13acq.crl DMSO {C:\NMR} cjsgrp 22
174.53
154.16
143.52
140.42
139.79
139.40
139.10
129.38
128.13
127.77
127.06
127.02
123.84
122.63
62.75
52.69
42.08
41.78
28.94
f1 (ppm)

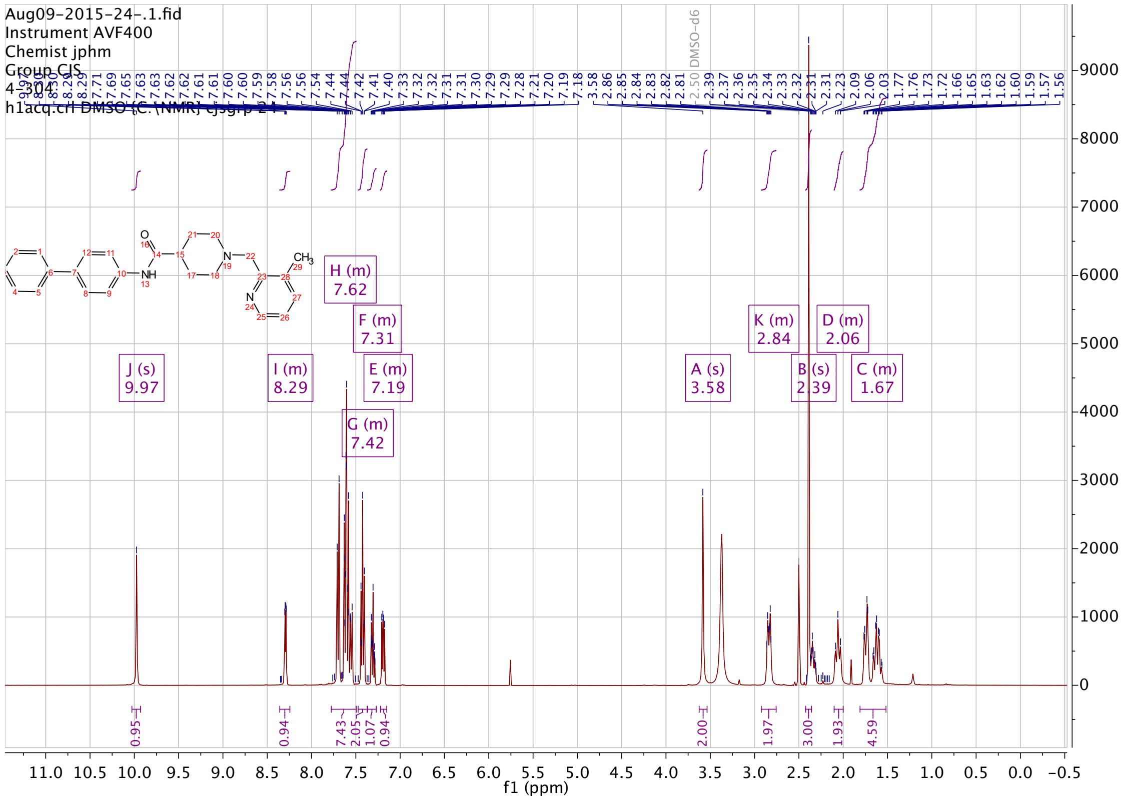
Aug09-2015-24-.1.fid
Instrument AVF400
Chemist jphm
Group CJS
4-304
h1acq.crl DMSO {C:\NMR} cjsgrp 24
J (s) 9.97
I (m) 8.29
H (m) 7.62
F (m) 7.31
E (m) 7.19
G (m) 7.42
A (s) 3.58
K (m) 2.84
B (s) 2.39
D (m) 2.06
C (m) 1.67
f1 (ppm)

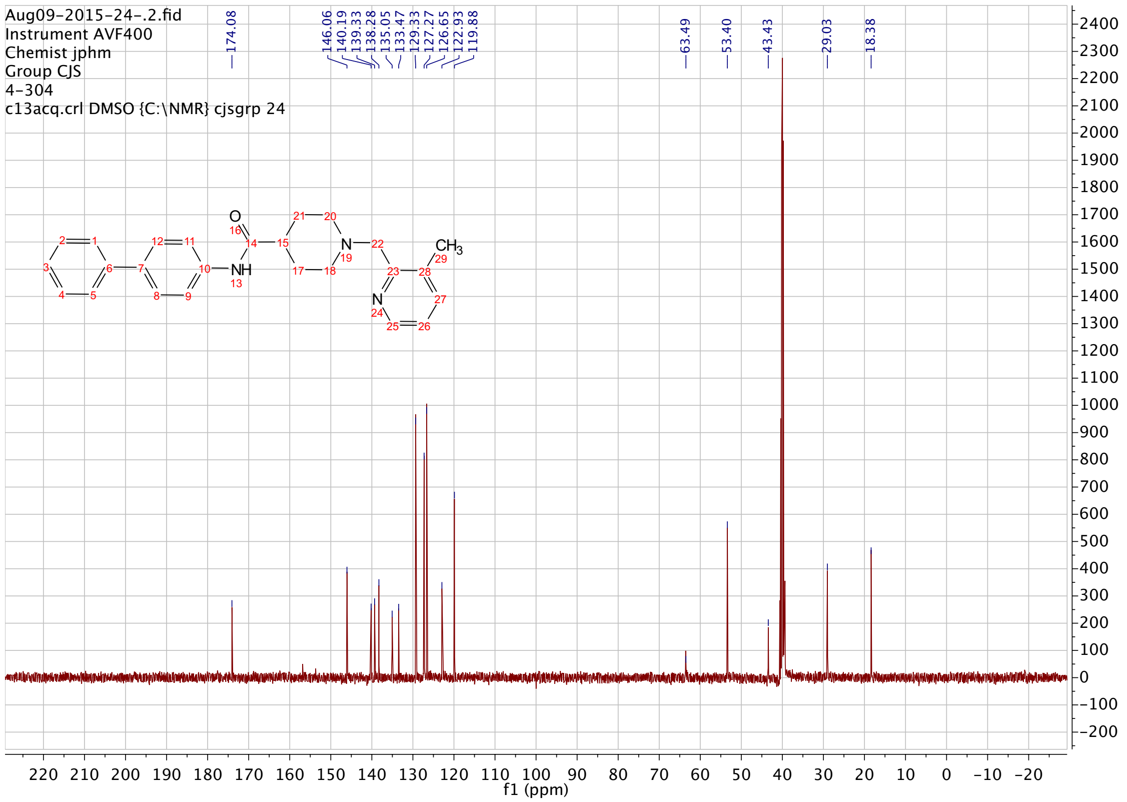
Aug09-2015-24-.2.fid
Instrument AVF400
Chemist jphm
Group CJS
4-304
c13acq.crl DMSO {C:\NMR} cjsgrp 24
174.08
146.06
140.19
139.33
138.28
135.05
133.47
129.33
127.27
126.65
122.93
119.88
63.49
53.40
43.43
29.03
18.38
f1 (ppm)

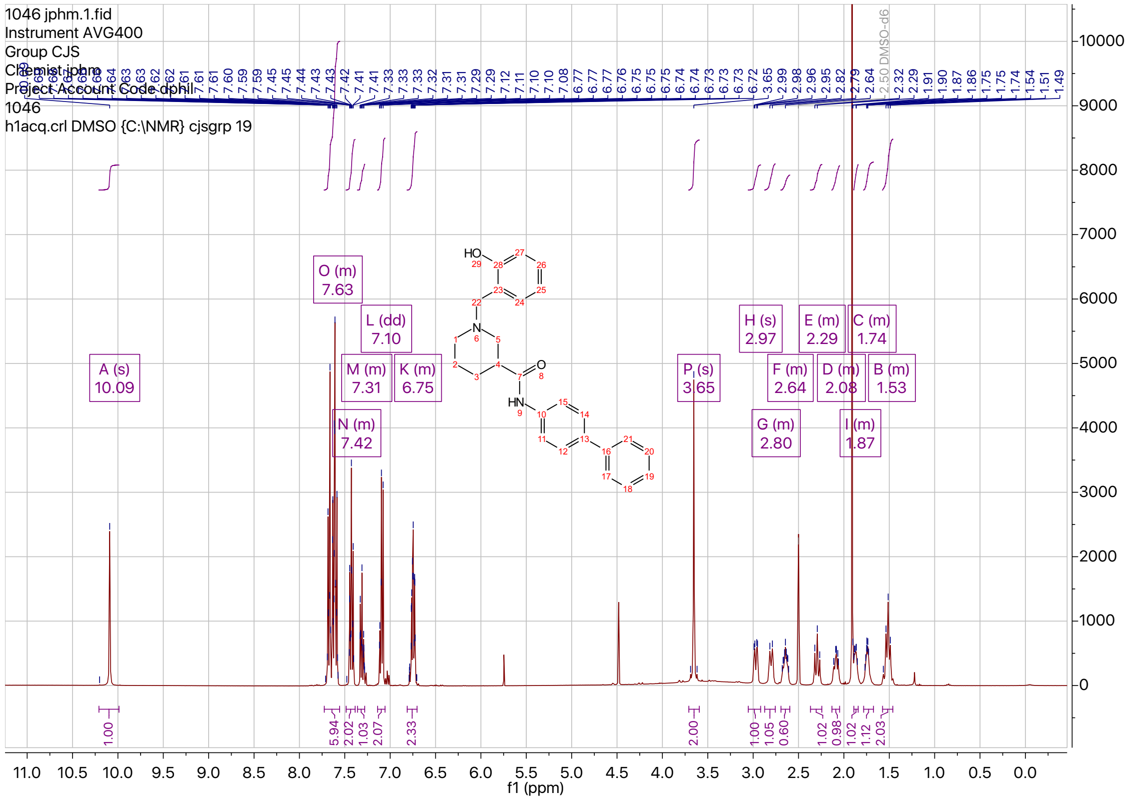

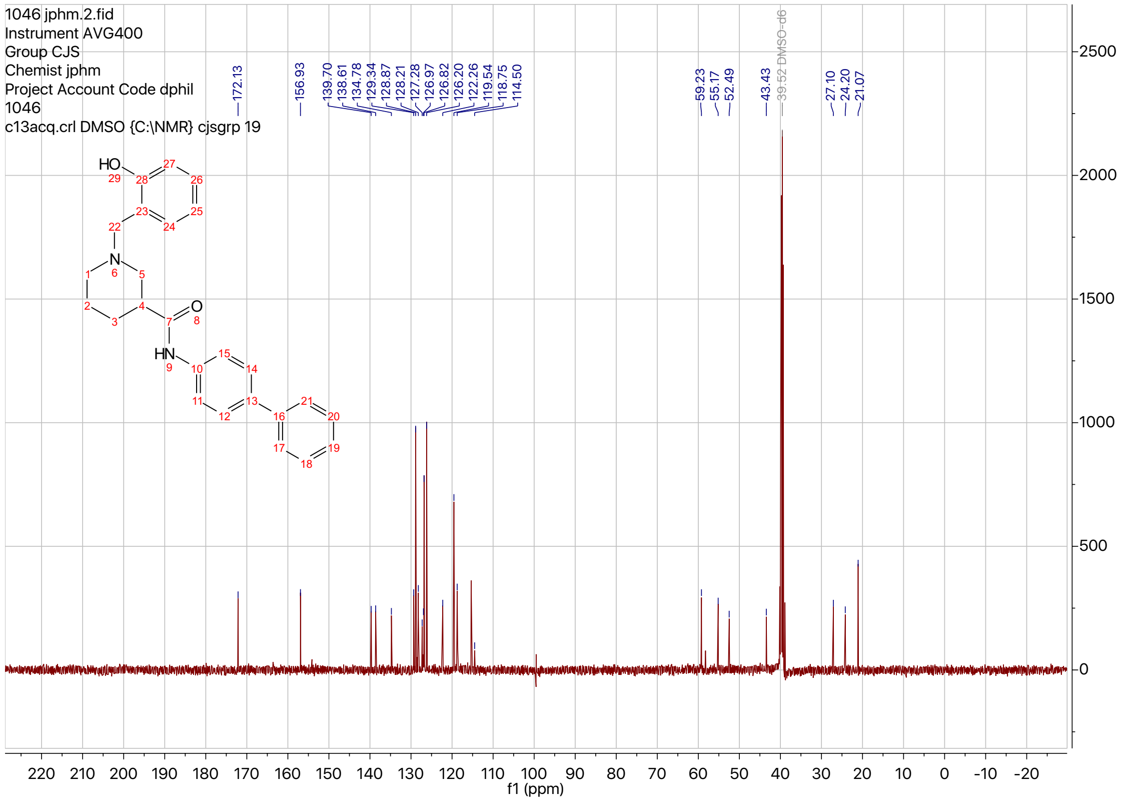

References

1. G. Deng, B. Zhao, Y. Ma, Q. Xu, H. Wang, L. Yang, Q. Zhang, T. B. Guo, W. Zhang, Y. Jiao, X. Cai, J. Zhang, H. Liu, X. Guan, H. Lu, J. Xiang, J. D. Elliott, X. Lin and F. Ren, *Bioorg Med Chem*, 2013, **21**, 6349-6358.
2. T. L. Yeh, T. M. Leissing, M. I. Abboud, C. C. Thinnes, O. Atasoylu, J. P. Holt-Martyn, D. Zhang, A. Tumber, K. Lippl, C. T. Lohans, I. K. H. Leung, H. Morcrette, I. J. Clifton, T. D. W. Claridge, A. Kawamura, E. Flashman, X. Lu, P. J. Ratcliffe, R. Chowdhury, C. W. Pugh and C. J. Schofield, *Chem Sci*, 2017, **8**, 7651-7668.
3. M. C. Chan, J. P. Holt-Martyn, C. J. Schofield and P. J. Ratcliffe, *Mol Aspects Med*, 2016, **47-48**, 54-75.
4. R. Chowdhury, I. K. Leung, Y. M. Tian, M. I. Abboud, W. Ge, C. Domene, F. X. Cantrelle, I. Landrieu, A. P. Hardy, C. W. Pugh, P. J. Ratcliffe, T. D. Claridge and C. J. Schofield, *Nat Comm*, 2016, **7**, 12673.
5. L. A. McNeill, E. Flashman, M. R. Buck, K. S. Hewitson, I. J. Clifton, G. Jeschke, T. D. Claridge, D. Ehrismann, N. J. Oldham and C. J. Schofield, *Mol Biosyst*, 2005, **1**, 321-324.
6. G. Winter, D. G. Waterman, J. M. Parkhurst, A. S. Brewster, R. J. Gildea, M. Gerstel, L. Fuentes-Montero, M. Vollmar, T. Michels-Clark, I. D. Young, N. K. Sauter and G. Evans, *Acta Crystallogr D Struct Biol*, 2018, **74**, 85-97.
7. M. D. Winn, C. C. Ballard, K. D. Cowtan, E. J. Dodson, P. Emsley, P. R. Evans, R. M. Keegan, E. B. Krissinel, A. G. W. Leslie, A. McCoy, S. J. McNicholas, G. N. Murshudov, N. S. Pannu, E. A. Potterton, H. R. Powell, R. J. Read, A. Vagin and K. S. Wilson, *Acta Cryst D, Biol Crystallogr*, 2011, **67**, 235-242.
8. A. J. McCoy, R. W. Grosse-Kunstleve, P. D. Adams, M. D. Winn, L. C. Storoni and R. J. Read, *J Appl Crystallogr*, 2007, **40**, 658-674.
9. P. D. Adams, P. V. Afonine, G. Bunkoczi, V. B. Chen, I. W. Davis, N. Echols, J. J. Headd, L.-W. Hung, G. J. Kapral, R. W. Grosse-Kunstleve, A. J. McCoy, N. W. Moriarty, R. Oeffner, R. J.

Read, D. C. Richardson, J. S. Richardson, T. C. Terwilliger and P. H. Zwart, *Acta Cryst D*, *Biol Crystallogr*, 2010, **66**, 213-221.

10. P. Emsley and K. Cowtan, *Acta Cryst D*, *Biol Crystallogr*, 2004, **60**, 2126-2132.
